# A combined bioinformatics and experimental approach identifies RMI2 as a Wnt/β-catenin signaling target gene related to hepatocellular carcinoma

**DOI:** 10.1186/s12885-023-10655-2

**Published:** 2023-10-24

**Authors:** Hung-Wen Tsai, Shu-Wen Cheng, Chou-Cheng Chen, I-Wen Chen, Chung-Liang Ho

**Affiliations:** 1 Department of Pathology, National Cheng Kung University Hospital, College of Medicine, National Cheng Kung University, 138 Sheng-Li Road, Tainan City, 704 Taiwan; 2 Institute of Basic Medical Sciences, College of Medicine, National Cheng Kung University, Tainan, Taiwan; 3 Department of Business Management, CTBC Business School, Tainan, Taiwan; 4 Institute of Molecular Medicine, College of Medicine, National Cheng Kung University, Tainan, Taiwan; 5 Department of Medical Laboratory Science and Biotechnology, College of Medicine, National Cheng Kung University, Tainan, Taiwan

**Keywords:** RMI2, β-Catenin/TCF complex, Wnt target genes, Bioinformatics

## Abstract

**Background:**

The Wnt/β-catenin signaling pathway plays an important role in embryogenesis and tumorigenesis. In human cancer, abnormal activity of Wnt/β-catenin signaling pathway induces overexpressed of downstream genes, and initiate oncogene. There are several target genes known to be key players in tumorigenesis, such as c-myc, cyclin D1, MMPs or survivin. Therefore, identifying the target genes of Wnt/β-catenin signaling pathway is important to understanding Wnt/β-catenin-mediated carcinogenesis. In this study, we developed a combined bioinformatics and experimental approach to find potential target genes.

**Methods:**

Luciferase reporter assay was used to analyze the promoter activity of RMI2. WST1 cell proliferation assays and transwell assays were performed to determine the proliferation and migration capacities of RMI2 overexpressing or knockdown stable hepatic cells. Finally, xenograft experiments were performed to measure the tumor formation capacity in vivo.

**Results:**

The results showed that *RMI2* mRNA was upregulated after LiCl treatment and Wnt3a-conditioned medium in a culture of SK-hep-1 cell lines. A chromatin immunoprecipitation (ChIP) assay showed that the β-catenin/T cell-specific factor (TCF) complex binds to the putative TCF binding site of the *RMI2* promoter. We then found a TCF binding site at − 333/− 326 of the *RMI2* promoter, which is crucial for β-catenin responsiveness in liver cell lines. *RMI2* was overexpressed in hepatoma tissue and cell lines, and it promoted the migration and invasion of HCC cells. Moreover, *RMI2* upregulated the expression of epithelial-mesenchymal transition (EMT) markers and the Wnt3a/β-catenin-related genes, but silencing *RMI2* had the opposite effects. Notably, the expression of *RMI2* was positively correlated with the clinical data of HCC patients who had significantly shorter overall survival (OS) and disease-free survival (DFS) (Both: *P* < 0.05). In addition, a total of 373 HCC patients’ data from the Caner Genome Atlas project (TCGA) were used to validate our findings.

**Conclusions:**

Taking all these findings together, we determined that *RMI2* was a new target gene of the Wnt/β-catenin signaling pathway. We also found that *RMI2* promotes EMT markers, HCC cell invasion, and metastasis, which indicated that *RMI2* is a potential target for preventing or at least mitigating the progression of HCC.

**Supplementary Information:**

The online version contains supplementary material available at 10.1186/s12885-023-10655-2.

## Introduction

The Wnt/β-catenin signaling pathway is important in embryogenesis and in tumorigenesis in a variety of tissue types, including colon and liver [1–3]. In colorectal tumors, gene mutations in the Wnt pathway, especially the adenomatous *polyposis coli* (*APC*) gene, are the major drivers in 90% of cases [4, 5]. In hepatocellular carcinomas (HCCs), although *APC* or β-catenin mutations are present in only 20–30% of cases, nuclear β-catenin accumulates in 33–67% of cases [5–7]. Mutations of these Wnt pathway components all have the same consequences: β-catenin accumulates in the cytoplasm which is then transported into the nuclei. It is believed [8, 9] that through an interaction with DNA-binding factors, higher levels of β-catenin induce uncontrolled activation of downstream genes, some of which are involved in tumorigenesis. Wnt/β-catenin signaling operates through the cytosolic stabilization of β-catenin (*CTNNB1*). Deletion or loss of function of its negative regulators, such as APC, glycogen synthase kinase-3β (GSK3β), or axis inhibition proteins (AXINs), results in abnormal stabilization of β-catenin, which enters the nucleus and forms a complex with T-cell factor/lymphoid enhancer-binding factor (TCF/LEF) transcription factor. This complex then activates the expression of target genes, the dysregulation of which significantly affects tumorigenesis.

Many β-catenin/TCF target genes have been reported to be critical for carcinogenesis (http://www.stanford.edu/~rnusse/pathways/targets.html). Most of these genes have in their promoter one or more TCF/LEF-binding elements composed of a highly conserved consensus sequence: 5′-(A/T)(A/T)CAA(A/T)G-3′ [10]. During the last decade, therapies that target WNT-responsive gene products like *COX-2* and *VEGF* have been developed. Inhibiting *COX-2* can block cell proliferation, cell migration, metastasis, and angiogenesis, and it can lead to apoptosis [11]. *COX-2* inhibitors, such as celecoxib and rofecoxib, were approved by the Food and Drug Administration (FDA) to shrink and abate colorectal polyps in familial adenomatous polyposis (FAP) patients [12]. *Survivin* and *matrix metallopeptidase* (*MMP*)*-7* in particular have also shown consistent promise as prognostic markers. For example, *survivin* expression is absent in most nonmalignant tissue, but it is upregulated in many types of cancer. High *survivin* expression is associated with a poor prognosis in medulloblastomas [13], HCC [14], gastric cancer [15], lung cancer [16], and breast cancer [17].

Previously we performed a combined bioinformatics and experimental approach to identify novel oncofetal genes using data from expressed sequence tags (EST). Six thousand one hundred eighteen EST cDNA libraries were chosen and classified into “immature”, “mature”, and “tumorous” groups. The calculated frequencies of the AFP gene in each group (immature: 175; mature: 10; and tumorous: 108) were used as references to set the thresholds of bioinformatics analyses to select genes expressed in the “immature”, downregulated in the “mature”, and re-expressed in the “tumorous” groups. The analyses resulted in 29 genes with known functions and 44 genes with unknown functions at that time. Five of the 29 known genes were indeed oncofetal genes, including AFP, IMP-1, FGF18, CGB, and SOX1 [18, 19]. We further studied the 44 then unknown genes and discovered two novel oncofetal genes, LRRC16B [20] and Lin28B [21]. In addition to the expected oncofetal genes, five of the 29 known genes were target genes of the Wnt/β-catenin pathway, including FGF18, Sox2, Axin2, BMP4, and EPHB3, probably because the Wnt/β-catenin pathway is important in both embryogenesis and carcinogenesis. This suggested that we may also find novel Wnt/β-catenin target genes in the unknown genes. We therefore modified the bioinformatics analyses using a group of known Wnt/β-catenin target genes to set the selection parameters, and perform experiments to verify the potentially novel target genes.

## Materials and methods

### Bioinformatic analysis

**Fig. 1:**
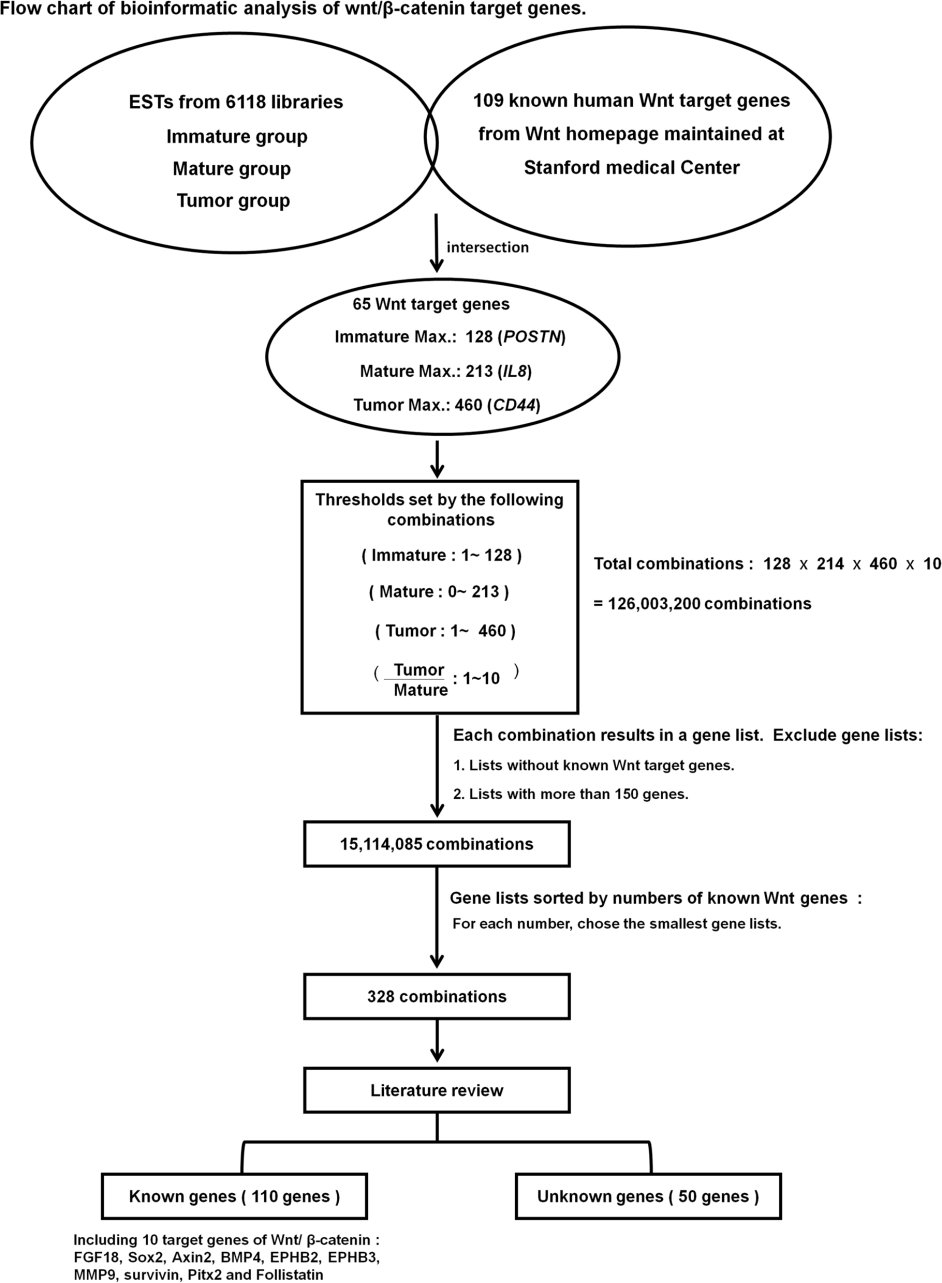
Flowchart of bioinformatic analysis of Wnt pathway target genes

As described previously, our laboratory performed bioinformatics analyses using the EST cDNA library data maintained by the Cancer Genome Anatomy Project (http://cgap.nci.nih.gov/) [20]. To classify each library, a certified pathologist read each library’s description, which contained its tissue origin, developmental stage (which included ‘fetus’, ‘infant’, ‘adult’ and ‘not specified’), and its histology or disease status (‘normal’, ‘tumor’, ‘other diseases’ and ‘not specified’). Totally 6118 libraries were divided into three groups: immature group (*n* = 483) from fetal tissue, infant tissue, placenta and cord blood；tumorous group (*n* = 3911) from benign and malignant tumors；and mature group (*n* = 1724) including those without information about age or disease status. Gene expression information was obtained from the file Hs_ExprData.dat, containing UniGene cluster ID, library ID and frequency. For example, the calculated frequencies of the AFP gene in each group of the libraries (immature group: 175; mature group: 10; and tumorous group: 108) were used to determine a set of thresholds for the bioinformatics analyses of novel oncofetal genes in our previous study [20]. In this study, we used a group of known target genes in the Wnt pathway to determine the ranges of parameters for various combinations of thresholds for the bioinformatics analyses. Of the 109 human target genes in the Wnt pathway (<https://web.stanford.edu/~rnusse/pathways/targets.html>), 65 were present in our expressed sequence tag (EST) database from the 6118 cDNA libraries (supplementary Table 1). The calculated frequencies of the 65 Wnt target genes in each were used to set the ranges for possible new Wnt targets. The highest frequency of the 65 genes in each group defined the upper limit of the bioinformatics analysis, and the lower limit was either 1 or 0. The resulting ranges were: immature group (1 to 128), mature group (0 to 213), and tumorous group (1 to 460). The range of the tumorous: mature ratio was 1–10. These ranges determined the combinations used to select genes from the 6118 EST libraries. For example, a combination of “fetal ≥ 5; mature ≤ 6; tumor ≥ 7; tumor/mature ≥ 8” would select for genes with frequencies ≥5 in the immature group, ≤ 6 in the mature group, ≥ 7 in the tumorous group, and with a tumor/mature ration ≥8. We finally used an exhaustive method to select better combinations. There are 126,003,200 combinations (128 × 214 × 460 × 10), and each gives rise to a gene list. Combinations that had no known Wnt target gene were discarded because they were unlikely to have new Wnt target genes in their corresponding gene lists. To ensure that the number of genes was manageable, combinations that yielded more than 150 genes were also excluded. The resulting 15,114,085 combinations were sorted based on the number of the 65 known Wnt target genes included in each corresponding list. For each number of known target genes, the smallest gene lists were selected because they had high percentages of known target genes and were likely to have new ones. Finally, we selected 328 gene lists that contained a total of 161 different genes (different gene lists shared some genes) (Fig. 1).

### Search for consensus TCF/LEF sites in candidate Wnt pathway target genes

From the Ensembl Genome Browser Database (https://asia.ensembl.org/index.html), we obtained sequences 2000 bp upstream of the transcription start sites of the *RMI2* gene. The putative binding sites were predicted using ALGGEN Promo (http://alggen.lsi.upc.es).

### Conditioned medium

To collect the conditioned medium (CM) from cultures of L cells that produce Wnt-3a (designated “Wnt3a-CM”), we seeded L cells (1 × 10^6^ cells in a 96-mm dish) and cultured them for 4 days. We then harvested the CM, centrifuged it at 1000×*g* for 10 min, and filtered the supernatant through a nitrocellulose membrane. Control CM was prepared from L cells that had been transfected with phosphoglycerine kinase (PGK)-neo only (designated “L-CM).

### RNA extraction and reverse transcriptase-polymerase chain reaction

The total RNA of cell lines was extracted using a reagent (Trizol) in accordance with the manufacturer’s protocols. Two micrograms of RNA was transcribed using reverse transcriptase (SuperScript II). The semiquantitative reverse transcriptase-polymerase chain reaction (RT-PCR) primer sequences are shown in Supplementary Table 2A. Original images of agarose gel are included in Supplementary Fig. 5.

### Western blotting

Collected cells were dissolved using a lysis buffer on ice and then centrifuged at 10,000×*g*, 4 °C for 20 min. Proteins were separated using 12% SDS-PAGE and then transferred to a PVDF membrane. Primary antibodies included mouse anti-β-catenin (BD Transduction Laboratories), rabbit anti-RMI2 (Abnova), rabbit anti-E-cadherin (Cell Signaling Technology), rabbit anti-vimentin (Cell Signaling Technology), rabbit anti-TWIST (Genetex), mouse anti-cyclin-D1 (Arigo), rabbit anti-c-Myc (Genetex), and mouse anti-β-actin (Millipore). To hybridize each marker separately, blots were cut based on the molecular weight of the proteins prior to hybridization with primary antibodies. Original blots with membrane edges are included in Supplementary Fig. 4.

### Retroviral infection

pMSCVpuro, pMSCV-RMI2, and pSUPERretro vectors were co-transfected into GP2–293 T packaging cells with VSV-G plasmids using the calcium phosphate method for 48 h. HCC cells were seeded in a 6-cm dish and cultured overnight in a 5% CO_2_ incubator at 37 °C. Retroviral supernatant was added to 8 ng/mL of polybrene and used to infect HCC cells. Pooled HCC cells expressing either pMSCVpuro or pMSCV-RMI2 were selected using 2 μg/mL of puromycin.

### Small hairpin RNA lentivirus

pLKO.1 lentiviral plasmids that expressed small hairpin RNA (shRNA) were purchased from the Taiwan National RNAi Core Facility (Academia Sinica, Taipei, Taiwan). Lentivirus particles were produced by the RNAi Core Facility in the Research Center of Clinical Medicine, National Cheng Kung University Hospital (NCKUH). To knock down *β-catenin* and *RMI2* expression, the shRNAs of *β-catenin*:

- (TRCN0000003844: 5′-CGCATGGAAGAAATAGTTGAA-3′;
- TRCN0000314920: 5′-GCTTGGAATGAGACTGCTGAT-3′;
- TRCN0000314990: 5′-ATCTGTCTGCTCTAGTAATAA-3′)

and of *RMI2*:

- (TRCN0000143095: 5′-CAGACCTTTCTGATAATCCCA-3′;
- TRCN0000144940: 5′-GAACTGGAGGTAGAAGATTTA-3′)

were used. A plasmid pLKO_TRC005 was the negative control.

### Chromatin immunoprecipitation assays

Chromatin immunoprecipitation (ChIP) assays were performed according to manufacturer’s instructions (Magna ChIP G Chromatin Immunoprecipitation; Millipore). Briefly, 3 × 10^6^ SK-hep-1 cells were stimulated for 3 h with 20 μM of LiCl, cross-linked with 1% formaldehyde for 10 min at room temperature, lysed and sonicated to average lengths of 200- to 1000-bps. After centrifugation, the samples were diluted 10x to assess input DNA. Anti-TCF-4 (Millipore), normal mouse immunoglobulin G (IgG) (Millipore), and 5 μg of monoclonal anti-β-catenin antibody (BD Transduction Laboratories) were added and incubated with gentle agitation overnight at 4 °C. The DNA-protein complexes were eluted and incubated with proteinase K at 62 °C for 2 h to reverse the formaldehyde cross-link. The primers used in the ChIP assays are listed in Supplementary Table 2B.

### Transfection and reporter gene assays

Transfection was done in triplicate using a reagent (Lipofectamin 2000). pRL-CMV plasmid was included as the internal control in every transfection mixture. The luciferase activity was normalized by renilla luciferase to adjust for variations in transfection efficiency. Cells were harvested 24 h after they had been transfected, and the luciferase activity was measured using a kit (Dual-luciferase assay; Promega).

### Plasmids and site-directed mutagenesis

*RMI2* promoter fragments were PCR-generated from human genome DNA using specific primers with restriction sites and then inserted into the luciferase reporter vector (pGL3-Basic; Promega). The mutations in the TBE sites of the *RMI2* promoter were generated using a kit (QuickChange site-directed mutagenesis; Stratagene, La Jolla, CA, USA). Primer sequences are shown in Supplementary Table 2C.

### Immunohistochemical staining

Five-micrometer-thick paraffined samples with malignant and non-malignant liver parenchyma tissue from each patient were histopathologically and immunohistochemically analyzed. Standard immunostaining procedures were used to determine *RMI2* and β-catenin. The samples were incubated with primary antibodies against *RMI2* (1:200) (Abnova) and β-catenin (Leica Microsystems GMBH: Taipei, Taiwan) overnight at 4 °C and washed in PBS. The slides were then treated with a streptavidin biotin immunoperoxidase complex (Envision^+^ kits, Dako, Glostrup, Denmark) for 1 h. The slides were lightly counterstained with hematoxylin to visualize immunoreactivity. Finally, the specimens were dehydrated in an ascending alcohol gradient.

### Immunofluorescence

Cells were fixed in 4% paraformaldehyde for 20 min. Cells were permeabilized with 0.1% Triton X-100 at 4 °C for 10 min. Primary antibody was diluted with phosphate buffered saline (PBS)/4% BSA for 4 °C overnight. After washing by PBS, the Alexa Fluor 555-conjugated secondary antibody was applied for 1 hour at room temperature. After counterstaining by DAPI, images were recorded on a fluorescence microscope.

### Co-immunoprecipitation (co-IP)

The lysate-antibody mixture was incubated overnight at 4 °C, then 30 μl of beads were added and the mixture was incubated for 3 hours at 4 °C in a tube rotator. The immunocomplexes were subsequently washed three times with 0.5 ml ice-cold PBS buffer and subjected to SDS-PAGE. The antibodies used for coimmunoprecipitation were: RMI2 (Abnova), Dvl-2 (Genetex), pGSK3β (Cell Signaling Technology) and β-catenin (BD Transduction Laboratories).

### WST-1 cell proliferation assay

Huh-7, PLC/PRF/5 stable pools (pMSCV, pMSCV-RMI2), and HepG2 and Hep3B stable pools (shRNA-RMI2–1, shRNA-RMI2-2, and shRNA-NC) were seeded in 24-well plates for 96 h. Triplicate wells were plated (1 × 10^4^ cells/well) for each time point (24-h intervals for 4 consecutive days).

### In vitro migration and invasion assay

Transwell migration and invasion assays were done in 24-well 8-μm-pore transwell plates in accordance with the manufacturer’s instructions (Corning, New York, NY, USA). 1 × 10^5^ cells in FBS-free DMEM were split into the upper chamber, where the transwell membrane was coated with (for invasion) or without (for migration) a cell culture matrix (Matrigel; BD Biosciences, San Diego, CA, USA). The lower chamber was filled with DMEM with 10% FBS. After 24 h culture, noninvasive cells on the upper side of the membrane were removed with a cotton swab. Each transwell was fixed by 4% paraformaldehyde, stained by crystal violet, and counted under a bright-field microscope for 5 randomly selected fields.

### Analysis using the cancer genome atlas data sets

The cBioPortal for Cancer Genomics (<https://www.cbioportal.org>) was used to access TCGA mRNA expression data. The provisional RNA-Seq V2 RSEM z-score for each gene was used. Three hundred seventy-three HCC cases had mRNA expression and OS data. The cBioPortal was used to assess the association of OS with the *RMI2* z-scores in TCGA data (using Kaplan-Meier survival analysis and the logrank test) using the R package on the cBioPortal for Cancer Genomics. Data were accessed from the cBioPortal between May 15 and July 15, 2017.

### Xenografted tumor model

Nod/SCID mice (age, 4–5 weeks) were purchased from National Laboratory Animal Center (NLAC) Taiwan in a standard pathogen-free environment laboratory. Mice were randomly divided into two groups: PLC/PRF/5-RMI2 and PLC-PMSCV-control. Cells were diluted and mixed with Matrigel (BD Biosciences, San Jose, CA) at a 1:1 ratio. The mixtures were subcutaneously injected into the left and right flanks of 6-week-old mice. The width and length of the tumors in each group were measured with vernier caliper twice a week. Tumor volume = (length × width^2^)/2. After 5 to 6 weeks, the mice were cervical dislocated under anesthesia and the tumors were isolated and weighted. Tumor tissues were confirmed by histopathological analysis. All mouse experiments were done in accordance with NCKU Laboratory Animal Care guidelines and were approved by the Institutional Animal Care and Use Committee in this study (IACUC #: 106129). The study was carried out in compliance with the ARRIVE guidelines, and all procedures for animal experiments followed the ethical standards.

### Statistical analysis

χ^2^ tests were used to compute the correlation coefficients of the immunostaining scores between two proteins. Significance was set at *P* < 0.05.

## Results

### Bioinformatics analyses

The bioinformatics analyses yielded a list of 161 genes (Fig. 1). Literature review showed that 111 of them had known functions; while the other 50 were novel. In the 111 known genes, we indeed found a high percentage of target genes of Wnt/β-catenin (10 among 111: FGF18, Sox2, Axin2, BMP4, EPHB2, EPHB3, MMP9, surviving, Pitx2, and Follistatin. Hence, we believed that the 50 unknown genes may contain novel target genes of Wnt/β-catenin. Experiments were performed as described below to evaluate these 50 genes and several potential Wnt target genes were found, including *RecQ-mediated genome instability protein 2* (*RMI2)*.

### Association between activated Wnt pathway and upregulated candidate targets

**Fig. 2:**
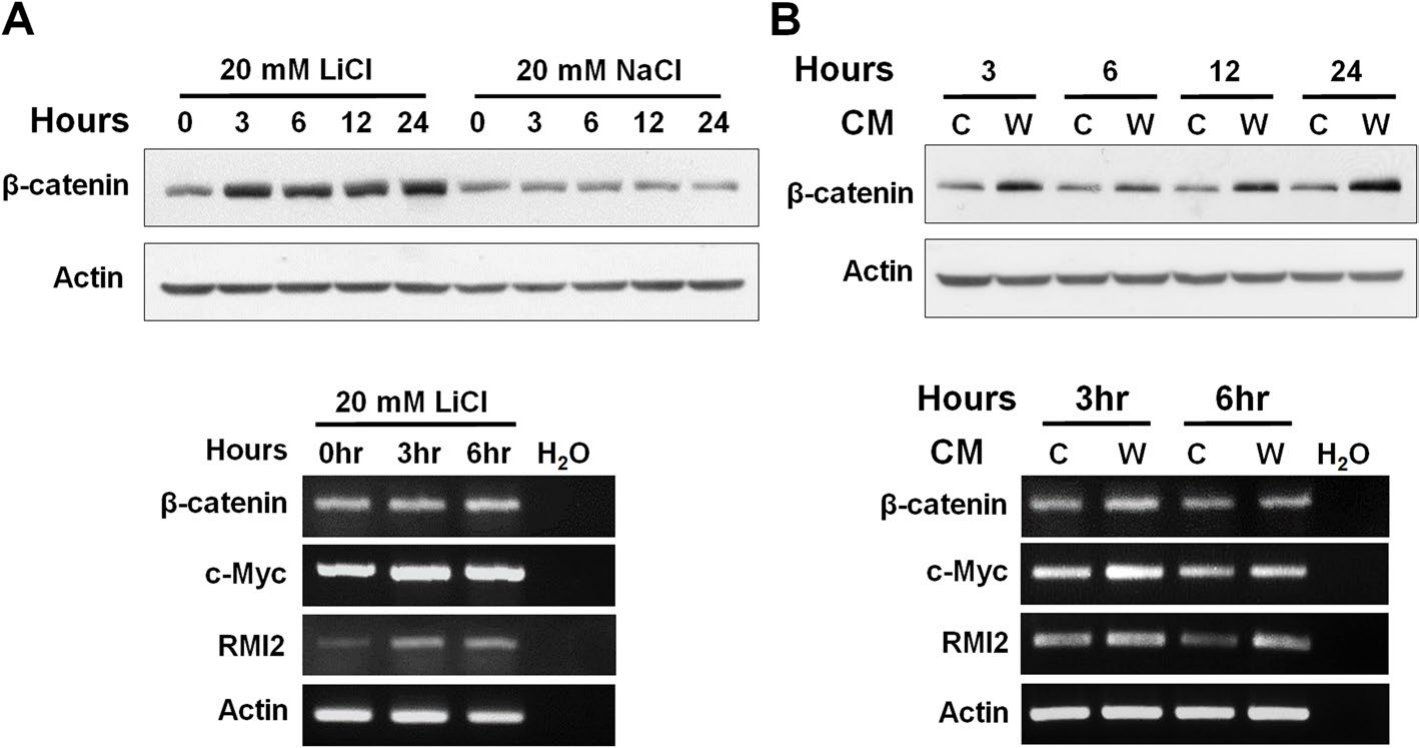
Activation of the canonical Wnt pathway in SK-hep-1 cells. **A** Effect of LiCl-induced Wnt/β-catenin signaling on the expression of candidate Wnt/β-catenin target genes. LiCl-treated SK-hep-1 cells were harvested at the indicated time points and analyzed for target gene expression using western blotting or RT-PCR. The β-catenin protein level was higher after LiCl treatment: it peaked at 3 h, but the mRNA level remained unchanged. **B** Wnt/β-catenin signaling was stimulated in SK-hep-1 cells with Wnt3a-CM (W) for different time periods (3–6 h) and compared with β-catenin accumulation in parental L-CM (C)-treated samples. c-MYC was a positive control for western blotting, and β-actin was the loading control for RT-PCR

We examined whether activating the Wnt pathway would affect candidate gene expression. Cells exposed to LiCl mimic the activated pathway by inhibiting glycogen synthase kinase (GSK3β), the Wnt pathway’s key kinase: it phosphorylates β-catenin and initiates its degradation. We treated SK-hep-1 (with a wild-type Wnt pathway) with LiCl for various time periods. β-catenin protein levels reached a maximum in 3 h, but the mRNA level did not change. This reflects the accumulation of β-catenin protein because of GSK3β inhibition and degradation. We also used Sk-hep-1 cells exposed for different time periods to 50% CM from L cells that expressed Wnt3a or from parental L cells. The protein level of β-catenin stabilized as early as 3 h after it had been stimulated with Wnt3a-CM, and it remained stabilized for up to 24 h (Fig. 2). By semi-quantitative RT-PCR of the 50 candidate genes, RMI2 mRNA was found to be induced at 3 h of treatment, when the levels of β-catenin were increased markedly, strongly suggesting that RMI2 expression can be regulated by β-catenin/TCF signaling in SK-hep-1 cell line.

### *RMI2* was a target of TCF/β-catenin signaling

**Fig. 3:**
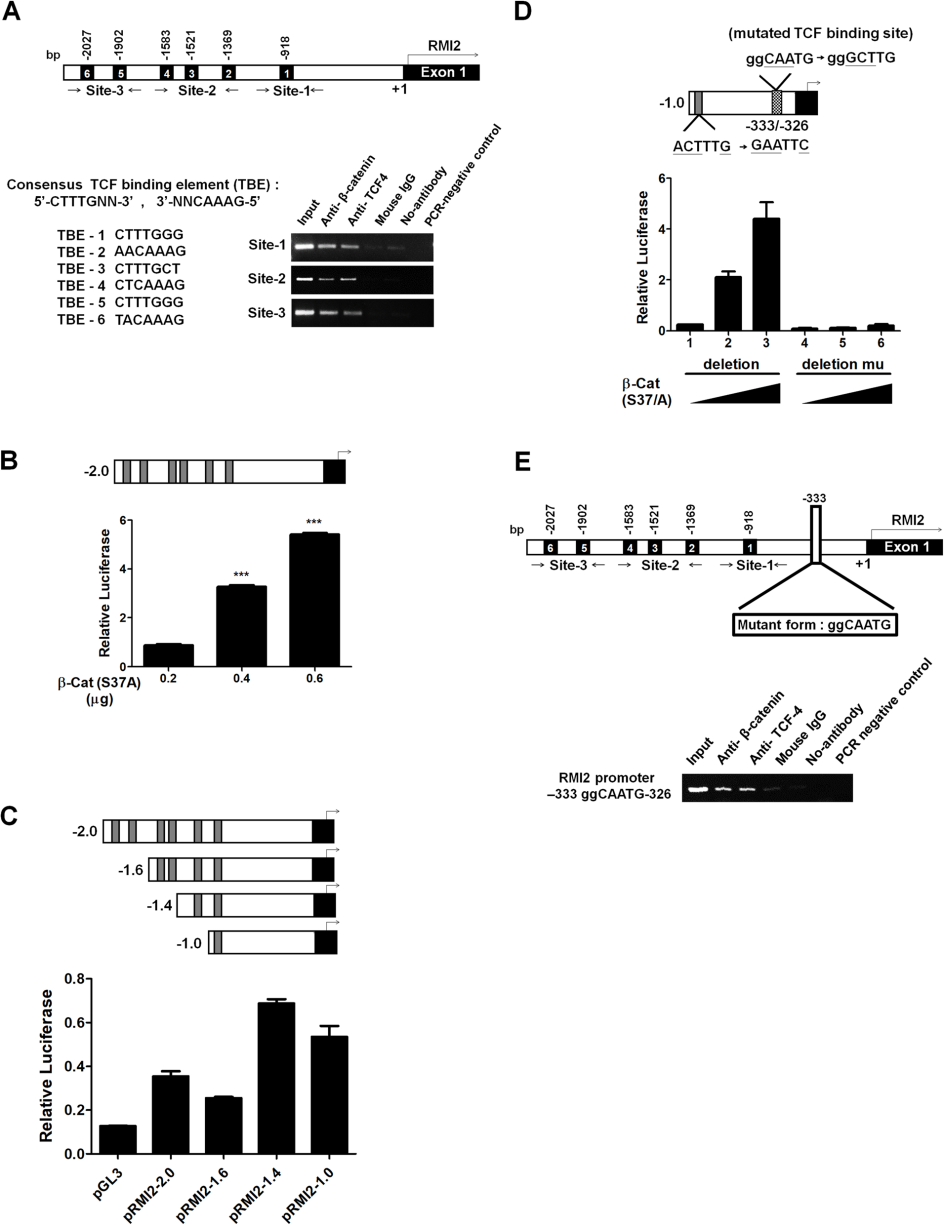
RMI2 was a direct target for β-catenin/TCF transcriptional regulation. **A** Schematic presentation of 6 potential TBEs in the RMI2 promoter. Arrows indicate the positions of primers used for the ChIP assay, which used IgG as a negative control, and used TCF4 or β-catenin monoclonal antibodies on DNA extracted from Sk-hep-1 cells. **B** The RMI2 promoter construct (pRMI2–2.0) was dose-dependently activated. **C** Schematic representation of pRMI2 deletion constructs. Gene reporter assays were done on a Huh-7 cell line transiently transfected with 0.1 μg of pRMI2 promoter constructs (pRMI2–2.0, pRMI2–1.6, pRMI2–1.4, and pRMI2–1.0) and 0.1 μg of empty vector. Ten nanograms of pRL-TK was co-transfected to normalize transfection efficiency. **D** Schematic representation of pRMI2–1.0 with double mutations. Single mutation of TBE-1 at − 924/− 918 did not significantly decrease β-catenin responsiveness (Supplementary Fig. 1). Additional site-directed mutagenesis of the atypical TBE at − 333/− 326 abolished β-catenin responsiveness. **E** ChIP assay was performed for the atypical TBE at − 333/− 326 using antibodies against β-catenin and TCF4

*RMI2* is a component of the BLM complex purified from cultured human cell lines. The deduced 147-amino acid protein has a single oligonucleotide-binding (OB)-fold domain. RMI2 has an apparent molecular mass of 18 to 20 kD (SDS-PAGE) [22, 23]. To examine whether β-catenin directly interacted with *RMI2* promoter, we analyzed the promoter and found at least six consensus TCF-binding elements (TBEs) (5′-CTTTGNN-3′ or 5′-NNCAAAG-3′, where N is A or T) within 2.0 kb upstream of the transcriptional start site [24]. The six TBEs are clustered in three sites. Using TCF4 and a monoclonal antibody that targets β-catenin, we did ChIP assays on the three sites using IgG as a negative control. All three sites were downregulated after they had been treated with β-catenin or TCF4 antibody (Fig. 3A). For subsequent reporter assays, we cloned the 2.0-kb fragment from the human *RMI2* promoter (pRMI2–2.0) and all 6 TBEs into the pGL3-basic vector fused to the luciferase gene. The Huh-7 cell line had a wild-type Wnt pathway with no accumulated endogenous β-catenin in the nuclei [25]. When RMI2 promoter was co-transfected with β-catenin (Ad-S37A) in Huh-7 cells, the luciferase expression level was significantly higher (Fig. 3B). Three RMI2 promoter constructs were generated to assess the contribution of TBEs to the transcriptional activity of the RMI2 promoter: pRMI2–1.6 (− 1650/− 53 bp); pRMI2–1.4 (− 1442/− 53 bp); and pRMI2–1.0 (− 1065/− 53 bp). The reporter activity of pRMI2–1.4, which includes two putative TBEs (TBE-1: − 924/− 918; TBE-2: − 1375/− 1369), was significantly improved after it had been co-transfected with β-catenin into Huh-7 cells (Fig. 3C). However, site-directed mutagenesis of the TBEs at − 924/− 918 or − 1375/− 1369 did not significantly affect the responsiveness of the RMI2 promoter to β-catenin (Supplementary Fig. 1). When a human promoter sequence has 80% homology to the known TCF/LEF-high mobility group (HMG) domain transcription factor consensus motif, it might be able to induce RMI2 promoter to become responsive to β-catenin; a mutation at this site would abolish β-catenin responsiveness, however [26]. We thus sequenced pRMI2–1.0 and found an element (− 333ggCAATG-326) that has 71% homology to the TCF consensus motif. Site-directed mutagenesis of the atypical TBE at − 333/− 326, in addition to TBE-1 at − 924/− 918, abolished β-catenin responsiveness (Fig. 3D). The DNA fragment around atypical TBE was pulled down by β-catenin and TCF4 antibodies in a ChIP assay (Fig. 3E).

### Correlation between RMI2 and clinicopathological features of HCC

We immunohistochemically stained for RMI2 expression in tissue samples from 60 cases of HCC and determined the resulting correlations with a variety of clinicopathological factors: patient age and gender, status of hepatitis virus infection, histological findings, tumor size, liver function, OS and DFS, and the IHC staining data of β-catenin. β-catenin was positively expressed in 16 (26.7%) cases, and RMI2 was positively expressed in 23 (38.3%) cases (Table 1). RMI2 expression was significantly correlated with satellite nodules (*P* = 0.024) and β-catenin expression (*P* = 0.043), but not with age, gender, tumor size, or differentiation. HBV infection (*P* = 0.066) and vascular invasion (*P* = 0.063) showed a tendency to be correlated with RMI2 expression. However, the mRNA expression level of β-catenin was not significantly correlated with that of RMI2 in TCGA HCC dataset. This finding suggested that the regulation of RMI2 may be based on subcellular localization of β-catenin protein in cytoplasm and nuclei rather than mRNA level (Supplementary Fig. 3 and Table 1).

**Table 1:** Relationship between RMI2 expression, clinicopathological features, and β-catenin aberration in 60 hepatocellular carcinomas (HCC)

Feature	***n***	RMI2 Overexpression (%)	***p-value***
**Age (years)**			**0.356**
**<60**	**32**	**14 (43.8)**	
**≧60**	**28**	**9 (32.1)**	
**Sex**			**0.271**
**Male**	**42**	**18(42.9)**	
**Female**	**18**	**5(27.8)**	
**Virus**			**0.291**
**HBV(-)HCV(-)**	**13**	**6(46.2)**	
**HBV(+)**	**28**	**13(46.4)**	
**HCV(+)**	**16**	**3(18.8)**	
**HBV(+)HCV(+)**	**3**	**1(33.3)**	
**Virus (only HBV or HCV)**			**0.066**
**HBV**	**28**	**13 (46.4)**	
**HCV**	**16**	**3(18.8)**	
**Non-cancerous tumor tissue**			**0.580**
**No cirrhosis**	**34**	**12(35.3)**	
**Cirrhosis**	**26**	**11(42.3)**	
**Tumor size**			**0.729**
**<5 cm**	**33**	**12(36.4)**	
**≧5 cm**	**27**	**11(40.7)**	
**Differentiation**			**0.226**
**Well**	**8**	**1 (12.5)**	
**Moderate**	**43**	**19(42.2)**	
**Poor**	**9**	**3(33.3)**	
**Multifocal tumor**			**0.553**
**No**	**50**	**20(40)**	
**Yes**	**10**	**3(30)**	
**Satellite nodules**			**0.024**
**No**	**48**	**15 (31.3)**	
**Yes**	**12**	**8(66.7)**	
**Encapsulated**			**0.191**
**No**	**50**	**21(42)**	
**Yes**	**10**	**2(20)**	
**Vascular invasion**			**0.063**
**No**	**30**	**8(26.7)**	
**Yes**	**30**	**15(50)**	
**Beta-catenin**			**0.043**
**Membranous**	**45**	**14(31.1)**	
**Nuclear and cytoplasmic**	**15**	**9(60)**	

### RMI2 expression was associated with overall survival and disease-free survival of HCC patients

**Fig. 4:**
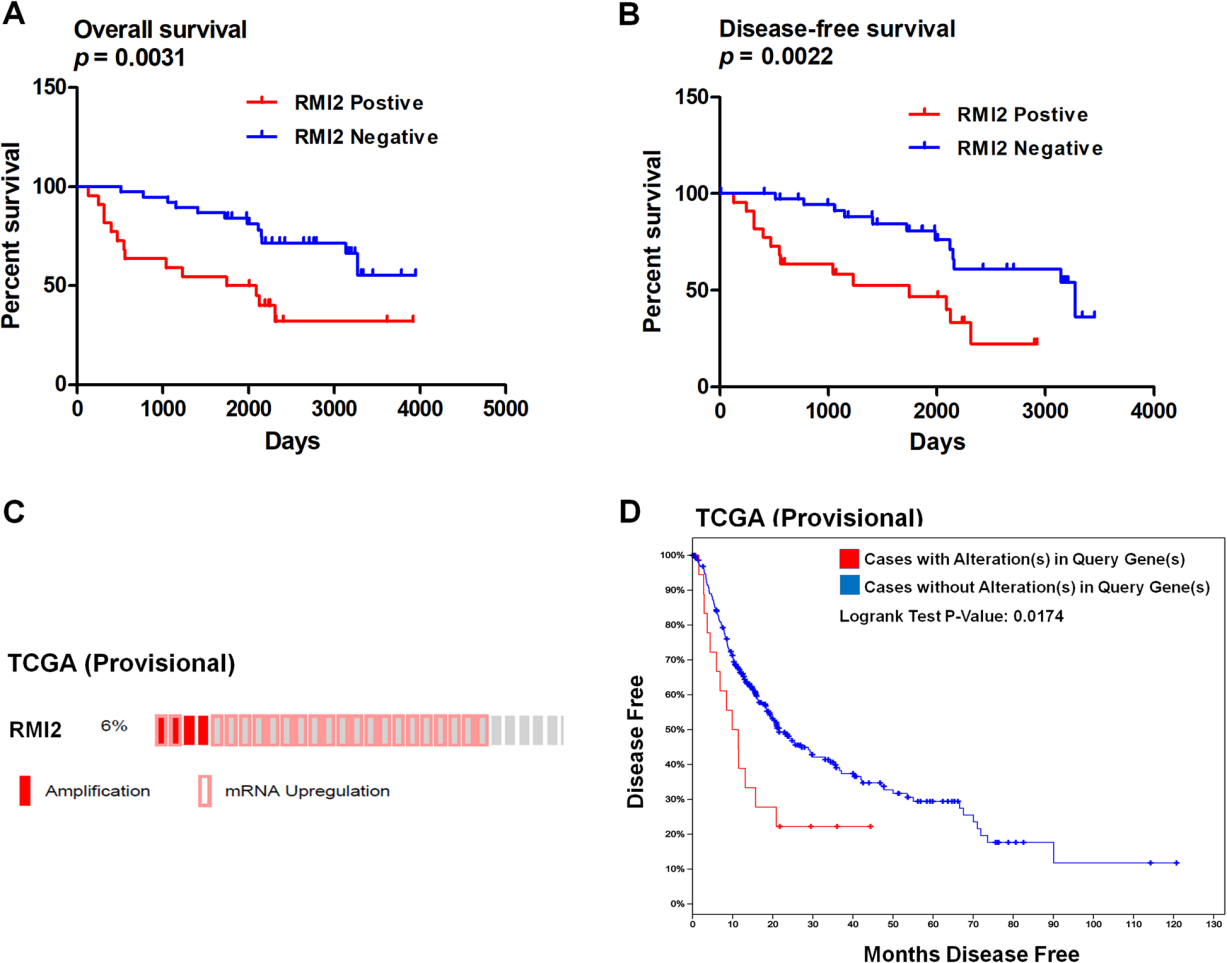
RMI2 and survival graphs of HCC. **A**-**B** Kaplan-Meier analysis showed that RMI2 was significantly associated with overall survival (OS) (*P* = 0.0031) and disease-free survival (DFS) (*P* = 0.0022) in 60 HCC cases with immunohistochemical staining. **C** “Oncoprint” analysis from the cBioPortal showed that 24 (6%) of 373 cases from the TCGA dataset had higher expression levels of the *RMI2* gene. **D** The higher level of *RMI2* expression was associated with a lower DFS in the TCGA cohort (*P* = 0.0174)

Kaplan-Meier analysis showed that RMI2 immunostaining levels were significantly (*P* < 0.05) negatively associated with OS (Fig. 4A) and DFS (Fig. 4B). Data from TCGA (The Cancer Genome Atlas) was used to validate these findings. Through cBioPortal for Cancer Genomics, 373 HCC cases from TCGA were found to have prognostic information. As shown in Fig. 4C, we found alterations (amplification and mRNA expression levels) of RMI2 in 6% (24/373) of the hepatic carcinomas analyzed. In addition, patients with RMI2 amplification had significantly poorer DFS compared to that with no alteration (*P* = 0.0174; Fig. 4D).

Furthermore, we downloaded data from TCGA and analyzed the available clinical parameters. We found that positive expression of RMI2 was significantly correlated with the lymph node metastasis (*p* = 0.048) and the AJCC stage (*p* = 0.0015), and no association was found between RMI2 expression and vascular invasion (Supplementary Fig. 2). These results suggest that expression of RMI2 predicts worse prognosis in patients with HCC.

### RMI2 and the Wnt/β-catenin/TCF signaling cascade

**Fig. 5:**
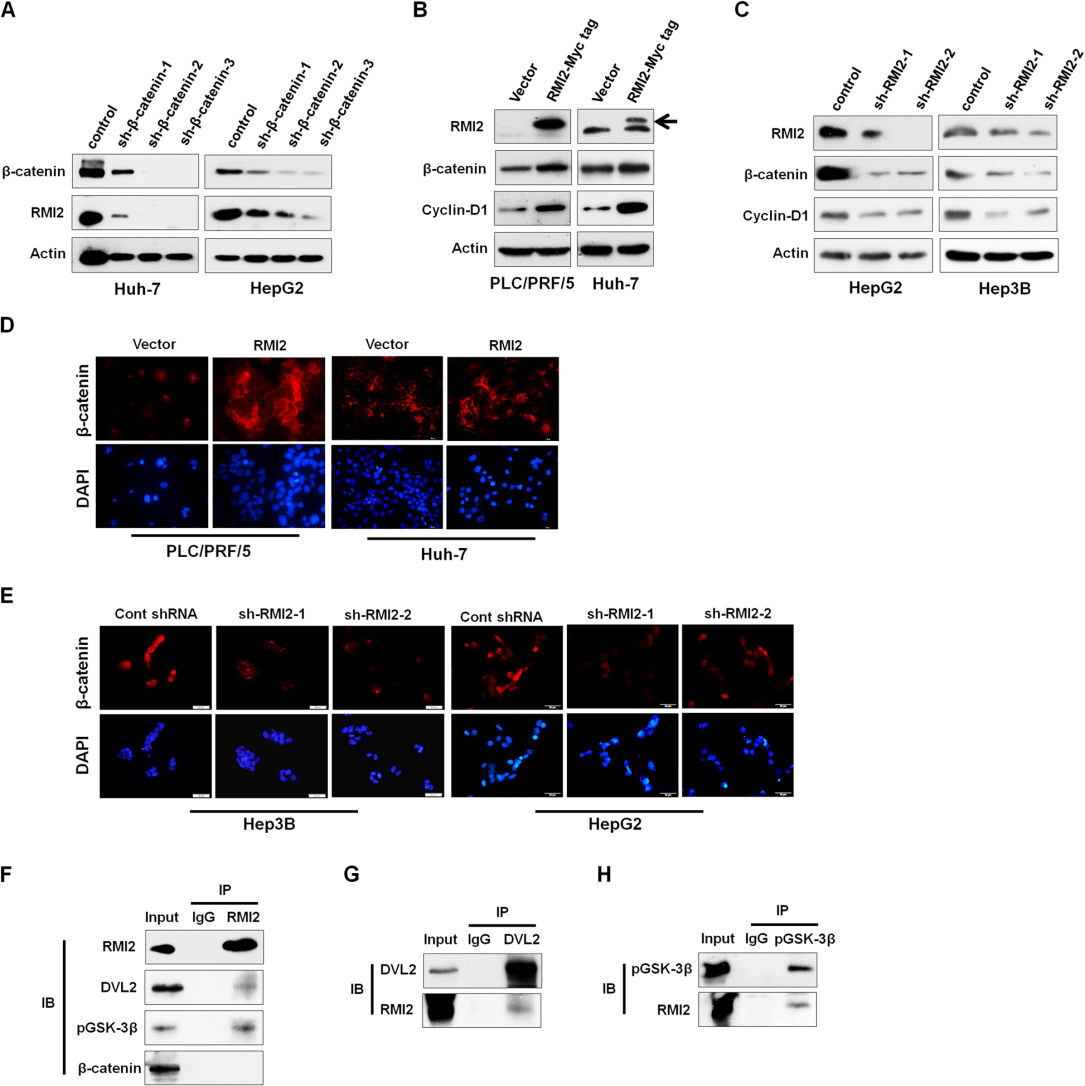
RMI2 activates the Wnt/β-Catenin/TCF signaling cascade. **A** Huh-7 and HepG2 cells transfected with β-catenin knockdown. **B**, **C** PLC/PRF/5, Huh-7, HepG2 and Hep3B cells were stably transfected with RMI2 and sh-RMI2, respectively. **D**, **E** Immunofluorescence assay for β-catenin in different groups of cells. Targeted proteins were stained in red color, and cell nuclei were counterstained with DAPI (blue). **F**, **G**, **H** Interaction between exogenous RMI2 with Dvl2 or GSK3β in PLC/PRF/5 cells. Western blotting of cell lysates subjected to co-IP with indicated antibodies against RMI2, Dvl2 or GSK3β

Gene-specific shRNA was used to knock down β-catenin expression in Huh-7 and HepG2 cells (Fig. 5A). RMI2 protein expression was positively dependent upon β-catenin expression-levels. We used western blotting to examine whether RMI2 regulated the Wnt pathway in HCC cell lines by testing Wnt/β-catenin’s downstream targets, like cyclin-D1. As shown in Fig. 5B, C, RMI2 overexpression triggered protein expression of β-catenin and cyclin D1 in PLC/PRF/5 and Huh-7 cells, but their expression in RMI2 knockdown (KD) cells was lower.

(HepG2 and Hep3B cells). By immunofluorescence staining, the increased nuclear staining of β-catenin upon RMI2 overexpression was observed, while RMI2 silencing had the opposite effect (Fig. 5D, E). The results suggested that RMI2 may act in a positive feedback loop on Wnt/β-catenin signaling in human HCC cells. Since the canonical Wnt signaling pathway is mediated by the Wnt/Frizzled-LRP6/Dvl2/Axin1-GSK3β-β-catenin complex, we hypothesized that RMI2 may relay Wnt signaling through the complex. As shown in Fig. 5F, G, H, exogenous RMI2 interacted with Dvl2 and GSK3β in PLC/PRF/5 cells, as evidenced by reciprocal co-IP assays. These results indicate that RMI2 may possibly interact with a complex containing DVL2 and GSK3β, leading to a positive feedback loop.

### RMI2 promotes cell proliferation in hepatic cancer cells

**Fig. 6:**
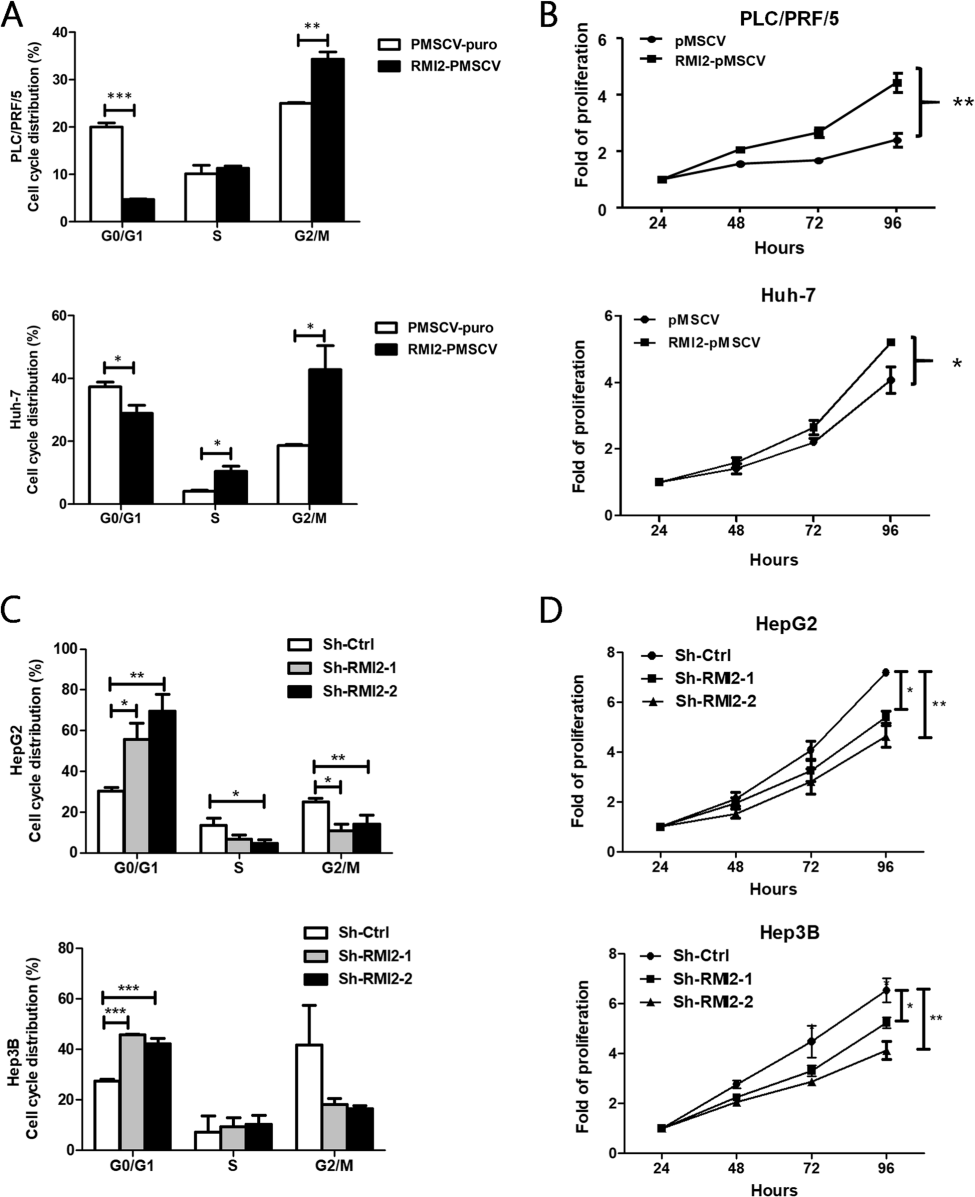
RMI2 modulated the proliferative ability of HCC cells in vitro. **A**, **C** Graph showing the cell cycle distribution status of each group by flow cytometry analysis. **B**, **D** Effect of RMI2 stable overexpression or knockdown on cell proliferation of HCC cells, as measured by WST1 assay in a 4-day culture period

Given that high RMI2 expression was significantly correlated with a poor prognosis, we then investigated the biological functions of RMI2 in HCC cells. Cell cycle phase distribution was determined by flow cytometry analysis. Figure 6A, C shows that RMI2 overexpression resulted in a substantial reduction in the G0–G1 phase and an increase in the number of cells in the G2-M phase, while RMI2 silencing had the opposite effect. Consistent with cell cycle results, cell proliferation assays showed that Huh-7 and PLC/PRF/5 cells was significantly higher when RMI2 was overexpressed and significantly lower in RMI2 KD HepG2 and Hep3B cells (Fig. 6B, D).

### RMI2 promotes EMT and modulates cell migration, and cell invasion in HCC

**Fig. 7:**
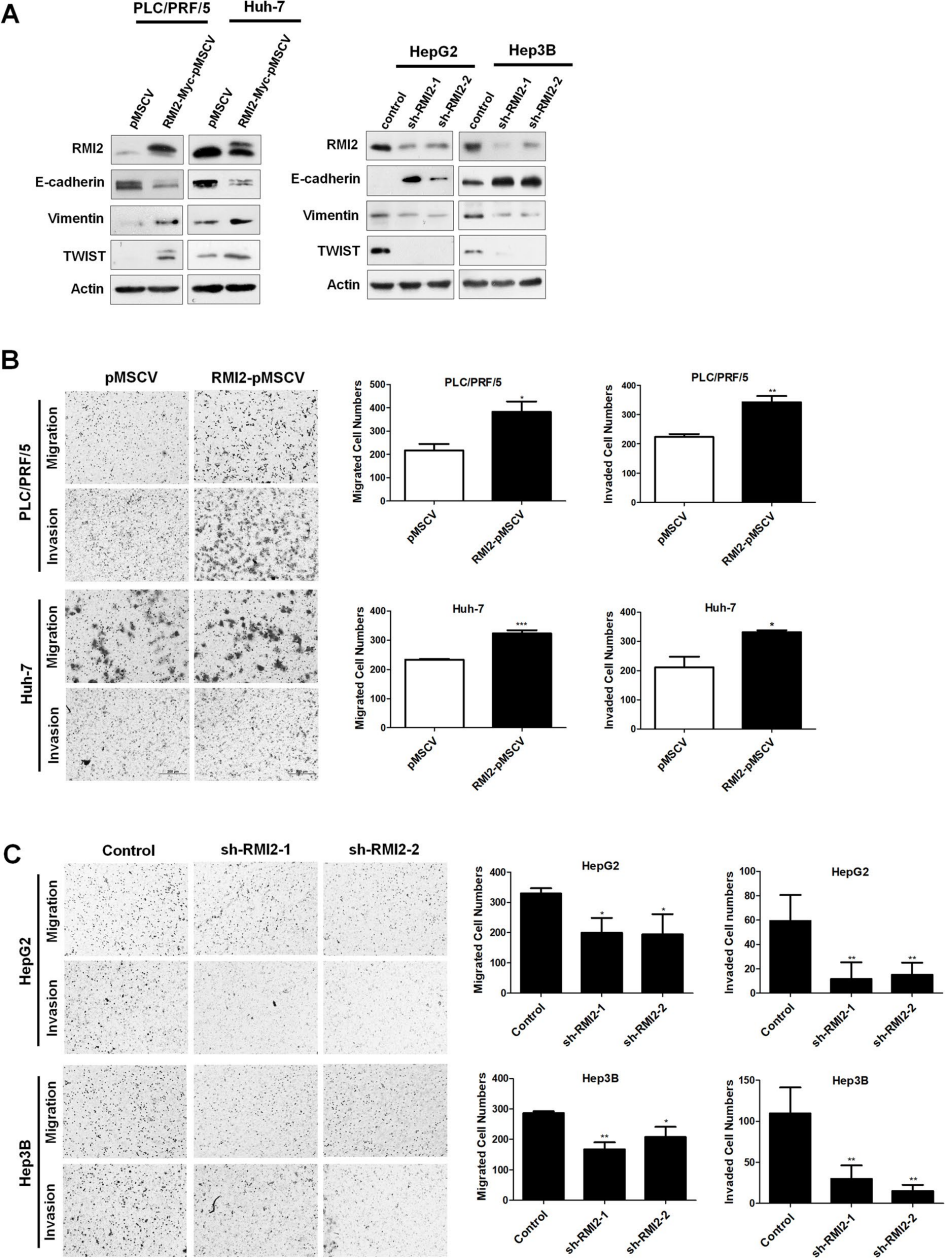
RMI2 modulated EMT induction cell migration and cell invasion of HCC lines. **A** Western blotting of the three EMT-related markers (E-cadherin, Vimentin and TWIST) was done in PLC/PRF/5 and Huh-7 cells transfected with an RMI2 expression plasmid and HepG2 and Hep3B cells transfected with RMI2 KD cells. **B**, **C** Migration and invasion of cells that stable RMI2 overexpressed or KD were measured using transwell assays. All values are expressed as mean ± SD from three independent experiments. Representative images of transwell chamber assays from three independent experiments are shown. **P < 0.01 versus empty vector

Aberrant activation of Wnt/β-catenin signaling reportedly contributes to the invasion, metastasis and progression of HCC [27]. To further investigate whether RMI2 affected the EMT process, we examined the expression levels of E-cadherin, vimentin and TWIST by western blot analysis. Western blotting showed that protein levels of vimentin and TWIST were significantly higher in cells that overexpressed RMI2 and significantly lower in RMI2 KD cells. Conversely, the expression levels of E-cadherin in RMI2-overexpressing cells decreased significantly while they increased in RMI2-silenced cells (Fig. 7A). Next we confirm the effect of RMI2 on the metastasis of hepatic cancer cells, in vitro transwell assays were performed either without any coating (for migration) or coated with Matrigel (for invasion). When endogenous RMI2 was inhibited, the number of cultured cells migrating or invading through the transwell decreased significantly compared with the control groups, while RMI2 overexpression had the opposite effects (Fig. 7B, C). Together, the results above reveal that RMI2 significantly modulated cell migration and invasion (*P* < 0.05).

### In vivo analysis of tumorigenesis

**Fig. 8:**
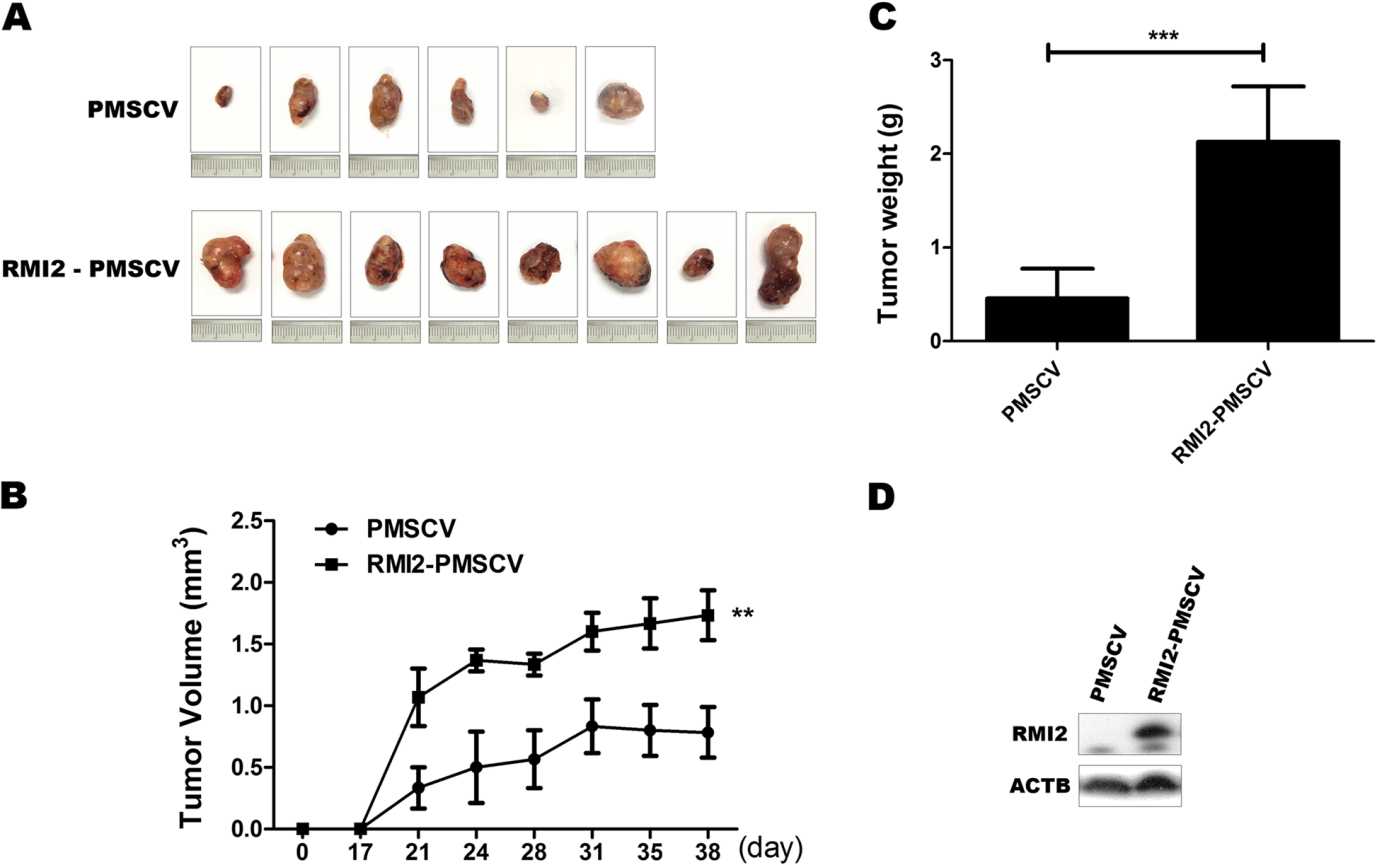
HCC progressed in vivo in mice that overexpressed RMI2. Nod-SCID mice were injected with PLC/PRF/5 RMI2-PMSCV cells (*n* = 4/group) or with control vector (*n* = 3/group). **A** Tumors isolated from control mice and from experimental mice. **B** Xenograft tumor growth curves. **C** Mean weights of xenografts tumors. **D** Expression levels of RMI2 protein in xenograft tumors were shown. The data were obtained in three independent experiments. All values are mean ± SD of three independent experiments; **P < 0.01

In order to test the tumor forming abilities of the stable PLC/PRF/5 cell lines in vivo, we performed Xenograft assays in SCID mice. Six to seven weeks after PLC/PRF/5 cells that overexpressed RMI2 had been injected into the flanks of the mice, the tumors formed by RMI2 overexpressing cells was significantly larger (Fig. 8A and B) (*P* < 0.01), and had higher mass (Fig. 8C) (*P* < 0.001) than the control tumors. Next, we examined RMI2 expression in the tumor samples with western blotting. As shown in Fig. 8D, RMI2 expression in the tumors formed with RMI2-overexpressing cells was higher than the control tumors. Taken together, the results in vivo were consistent with our results in vitro, which further confirmed that RMI2 played an important role in enhancing the tumorigenicity of hepatic cancer cells.

## Discussion

The canonical Wnt pathway is highly conserved and is involved in a variety of differentiation events during embryonic development, e.g., axis formation, cell proliferation, cell differentiation, and morphogenesis. β-catenin is considered the key molecule in this pathway. In addition to its functions in the early development of vertebrates, the Wnt pathway initiates tumor formation when aberrantly activated. These characteristics makes the pathway itself and its targets important foci of cancer research.

Previous studies have reported several Wnt target genes, such as TGFB induced factor homeobox 1 (TGIF1), Sry-related HMG box 9 (SOX9) and vasodilator-stimulated phosphoprotein (VASP), play in the positive feedback loop to support Wnt activity in various types of human malignancies [28–30]. In this study, RMI2 expression was increased by Wnt/β-catenin pathway activation in hepatic cancer cell lines; while reciprocally, β-catenin protein level was increased with RMI2 overexpression but decreased with RMI2 knockdown. By co-IP assays, we confirmed that RMI2 interact with a protein complex containing DVL2 and/or pGSK-3β. A positive correlation between RMI2 and β-catenin expression was also observed in HCC tissues. Taken all these together, it is conceivable that RMI2 may play in a positive feedback loop in the Wnt/β-catenin pathway, just like the afore mentioned Wnt/β-catenin targets.

*RMI2* was found to be essential for the BLM-Topo IIIa complex [22, 23] and association of *RMI2* with the BLM-Topo IIIa complex is through *RMI1* [22]. Mutations in *BLM*, a RecQ-like helicase, have been linked to the autosomal recessive cancer-prone disorder Bloom’s syndrome. *RMI2* was widely considered to play a crucial role in DNA repair surveillance, and prevents genomic instability [31]. *RMI2* was identified as significant cervical squamous cell carcinoma after expression validation and survival analysis [32]. *RMI2* was also reported to be associated with the worse prognosis in pancreatic cancer [33]. Recently, a study related *RMI2* expression to worse prognosis of lung cancer [34, 35]. Our data analysis implied that over-expression of *RMI2* was associated with worse OS in HCC patients. It remains unclear whether *RMI2* contributes to tumorigenesis through a BLM-related mechanism, or through dysregulation of the Wnt/β-catenin pathway which may not involve the BLM-Topo IIIa complex.

In Table 1, RMI2 was correlated with the aberrant accumulation of β-catenin in human HCC samples. In addition, we found that RMI2 expression was significantly correlated with satellite nodules in HCC patients. A study [36] that used transcriptome analysis to identify 6 subgroups of HCC associated with clinical and genetic characteristics reported that Group-6 tumors were characterized by satellite nodules and strongly related to β-catenin mutations that lead to Wnt pathway activation. This is consistent with our findings.

β-catenin is an important gene in HCC tumorigenesis and progression [7, 37–41], and the accumulation of nuclear β-catenin is correlated with the presence of gene mutations [7, 38–41]. There are conflicting reports about whether β-catenin mutations occur early or late in the development of HCC and about their association with the histological tumor grades [42–44]. Mutations in the β-catenin gene were believed to be associated with less aggressive tumors and were more frequently seen in earlier stages of HCC (stages I and II) [39, 45–47]. However, several studies [7, 38–40, 48] reported that the accumulation of β-catenin and that gene mutations were not associated with early stages in hepatocarcinogenesis but that they might be associated with the malignant progression of HCC and not with histological tumor grades. There are also several conflicting reports on the association of β-catenin gene mutations with tumor prognosis [40, 41, 49]: Some claim that β-catenin mutations are associated with greater expression of nuclear or cytoplasmic β-catenin [7, 38, 41, 42], that nuclear β-catenin expression is associated with a relatively good prognosis, and that cytoplasmic β-catenin expression is associated with a poorer prognosis [7, 38, 39]. Others claim that the accumulation of β-catenin in cytoplasm and the nucleus is significantly correlated with a worse prognosis of HCC [40, 50], which agrees with the survival data in our study.

## Conclusion

We established a bioinformatics analytical model and found RMI2 as a new direct target gene in the Wnt pathway*.* RMI2 is a worse prognostic biomarker for HCC. The molecular mechanisms of RMI2 in HCC cell aggressiveness might be associated with the dysregulation of the Wnt pathway.

### Supplementary Information

**Additional file 1: Supplementary Fig. 1.** Mutation study of RMI2 promoter. (A)Diagram of the mutation constructs, mutated sites are as indicated. The core sequence (CAAAG) located between positions-1369 and-1375 was mutated as (GCTAG). β-catenin (S37/A) can activate the mutation of deletion. Deletion and mutation of deletion were cotransfected with increasing amount of β-catenin (S37/A) expression construct (0.2, 0.4, 0.6 μg). (B) Diagram of the mutation constructs, mutated sites are as indicated. The core sequence (ACTTTG) located between positions − 918 and − 924 was mutated as (GAATTC). β-catenin (S37/A) can activate the mutation of deletion. Transfections were carried out as described in *A*.

**Additional file 2: Supplementary Fig. 2.** Clinical significance of RMI2. (A, B) RMI2 mRNA expression levels in HCC of different pathological stages and lymph node metastasis (*p* < 0.05).

**Additional file 3: Supplementary Fig. 3.** Correlation of RMI2 and beta-catenin (CTNNB1) expression in the TCGA dataset.

**Additional file 4: Supplementary Fig. 4.** The original images for western blot in article.

**Additional file 5: Supplementary Fig. 5.** The original images for agarose gel in article.

**Additional file 6.** Steps to download X-crunch dataset.

**Additional file 7: Supplementary Table 1.** Target genes of Wnt/beta-catenin signaling from http://www.stanford.edu/group/nusselab/cgi-bin/wnt/.

**Additional file 8: Supplementary Table 2.** (A) Primer sequence used for PCR assays. (B) Primer sequence used for ChIP assays. (C) Primer sequence used for site-directed mutagenesis assays.

## Data Availability

The informatics analyzed are available in OSFHOME database. Its hyperlink is https://osf.io/pf6hr/files/osfstorage. Its identifier is doi:10.17605/OSF.IO/PF6HR. Please follow the step description in supplementary Fig. 6 to download the X-crunch.txt or X-crunch.xlsx files. The datasets used and/or analyzed during the current study are available from the corresponding author on reasonable request.
