# Supplementary material for: A combined bioinformatics and experimental approach identifies RMI2 as a Wnt/β-catenin signaling target gene related to hepatocellular carcinoma
Source: BMC Cancer. 2023 Oct 24;23:1025. doi: 10.1186/s12885-023-10655-2 (PMC10594864; doi:10.1186/s12885-023-10655-2)
Supplement: Supplementary file 1 — Additional file 1: Supplementary Fig. 1. Mutation study of RMI2 promoter. (A)Diagram of the mutation constructs, mutated sites are as indicated. The core sequence (CAAAG) located between positions-1369 and-1375 was mutated as (GCTAG). β-catenin (S37/A) can activate the mutation of deletion. Deletion and mutation of deletion were cotransfected with increasing amount of β-catenin (S37/A) expression construct (0.2, 0.4, 0.6 μg). (B) Diagram of the mutation constructs, mutated sites are as indicated. The core sequence (ACTTTG) located between positions − 918 and − 924 was mutated as (GAATTC). β-catenin (S37/A) can activate the mutation of deletion. Transfections were carried out as described in A. [file 12885_2023_10655_MOESM1_ESM.pdf]

Supplementary Figure 1.

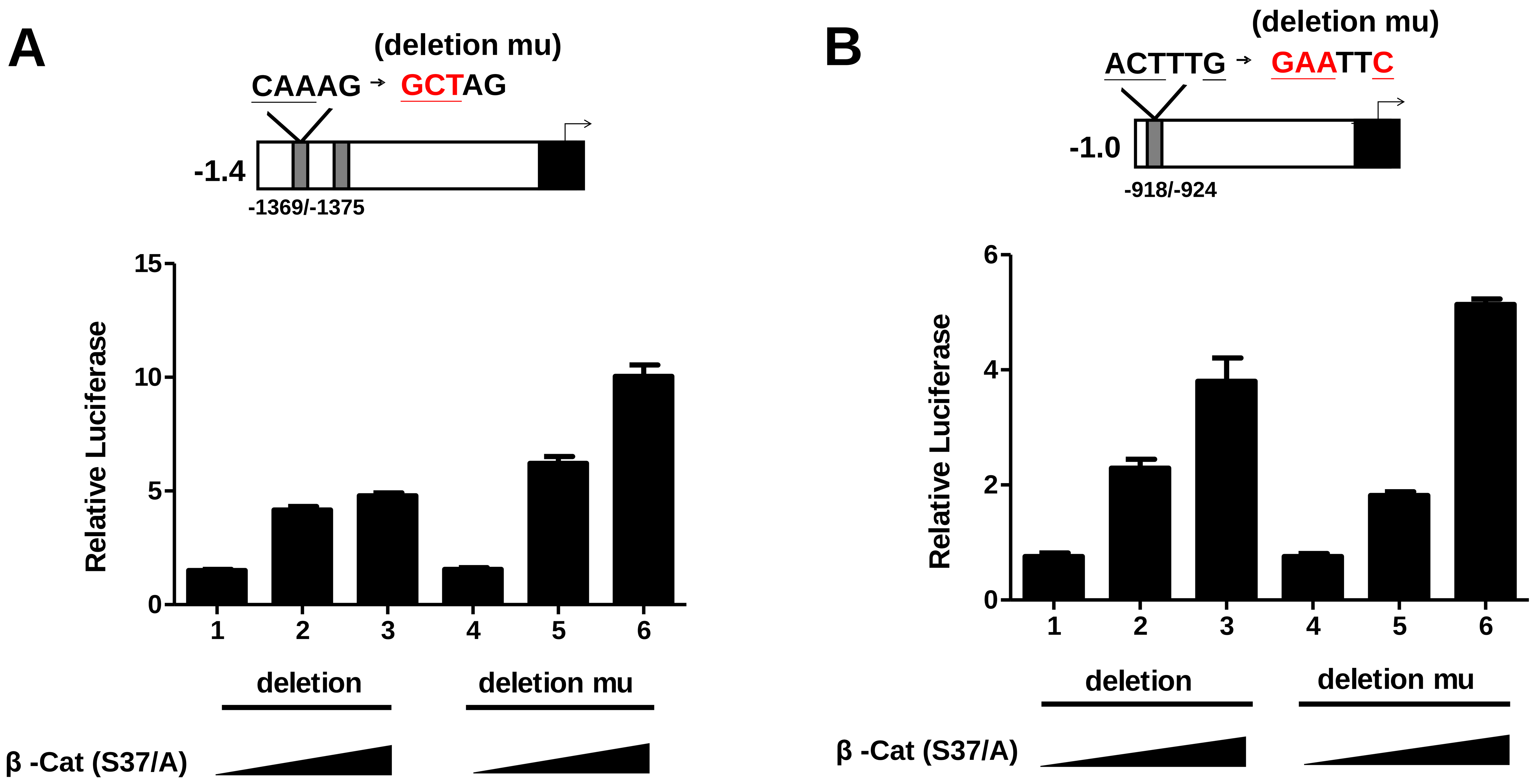

Mutation study of RMI2 promoter. (A) Diagram of the mutation constructs, mutated sites are as indicated. The core sequence (CAAAG) located between positions -1369 and -1375 was mutated as (GCTAG). β-catenin (S37/A) can activate the mutation of deletion. Deletion and mutation of deletion were cotransfected with increasing amount of β-catenin (S37/A) expression construct (0.2, 0.4, 0.6 μg). (B) Diagram of the mutation constructs, mutated sites are as indicated. The core sequence (ACTTTG) located between positions -918 and -924 was mutated as (GAATTC). β-catenin (S37/A) can activate the mutation of deletion. Transfections were carried out as described in *A*.
