# Supplementary material for: A combined bioinformatics and experimental approach identifies RMI2 as a Wnt/β-catenin signaling target gene related to hepatocellular carcinoma
Source: BMC Cancer. 2023 Oct 24;23:1025. doi: 10.1186/s12885-023-10655-2 (PMC10594864; doi:10.1186/s12885-023-10655-2)
Supplement: Supplementary file 2 — Additional file 2: Supplementary Fig. 2. Clinical significance of RMI2. (A, B) RMI2 mRNA expression levels in HCC of different pathological stages and lymph node metastasis (p < 0.05). [file 12885_2023_10655_MOESM2_ESM.pdf]

Supplementary Figure 2.

A

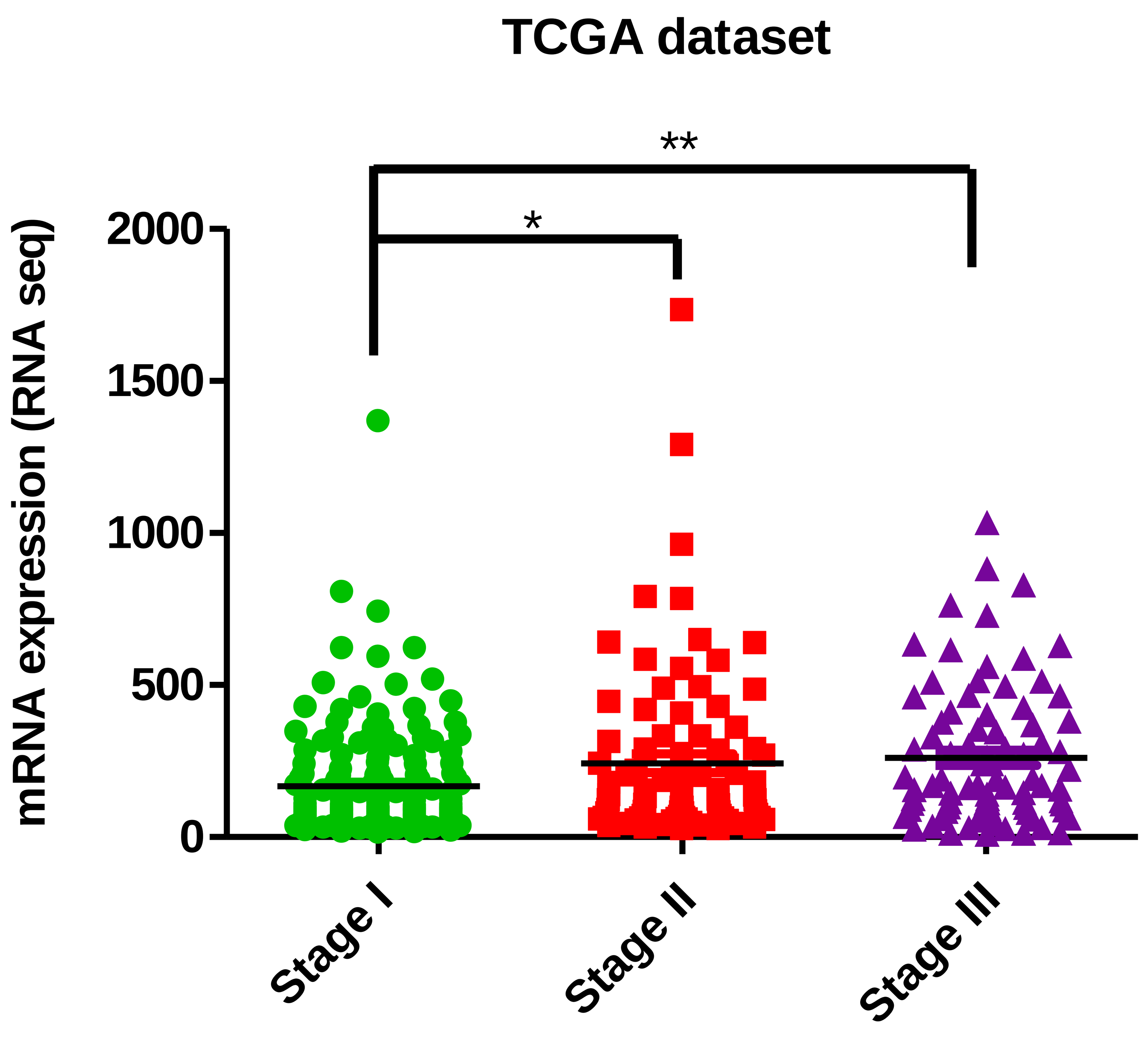

B

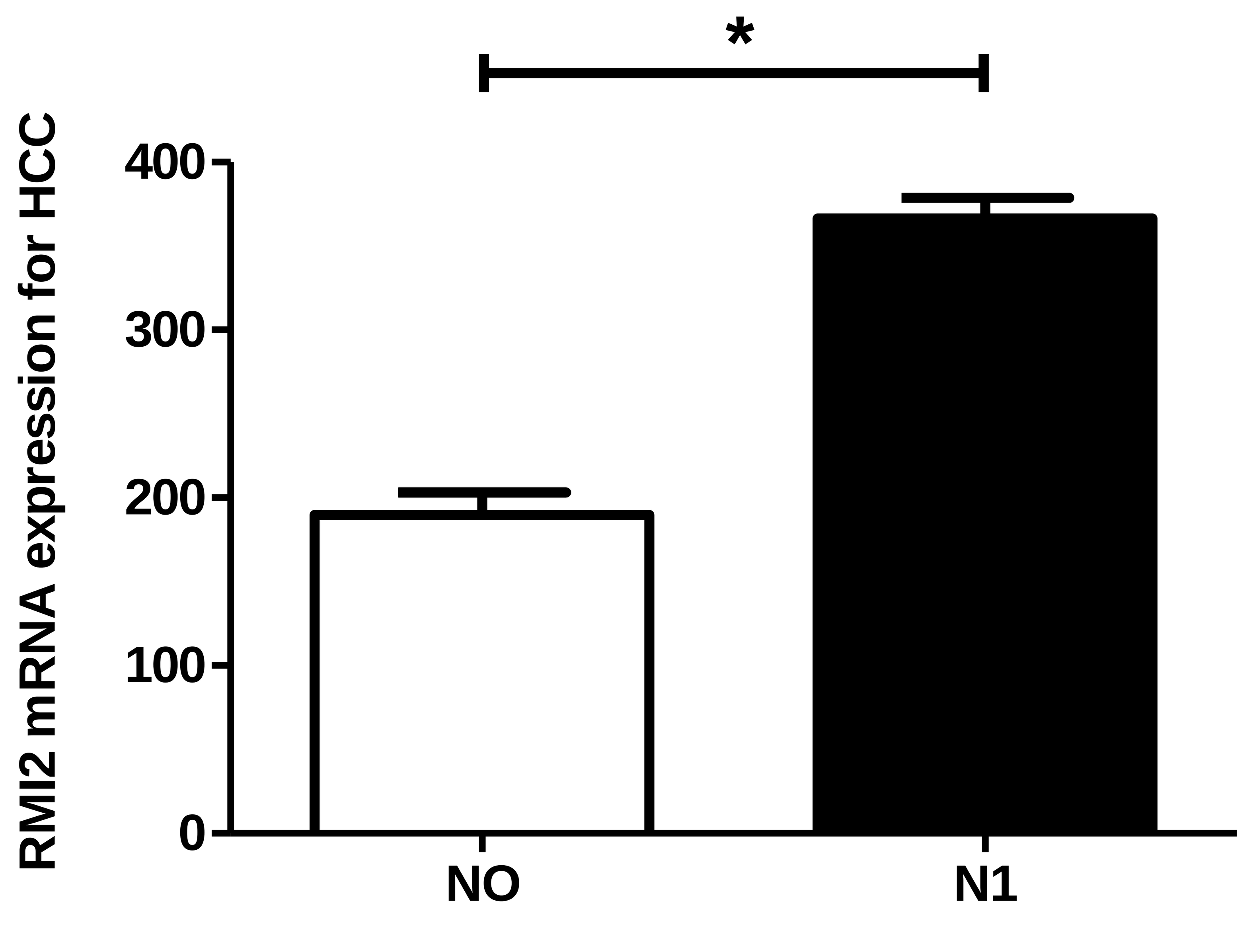

N0: No cancer was found in the lymph nodes
N1: The cancer has spread to one to three axillary lymph nodes

Clinical significance of RMI2. (A, B) RMI2 mRNA expression levels in HCC of different pathological stages and lymph node metastasis ($p<0.05$).
