# Supplementary material for: A combined bioinformatics and experimental approach identifies RMI2 as a Wnt/β-catenin signaling target gene related to hepatocellular carcinoma
Source: BMC Cancer. 2023 Oct 24;23:1025. doi: 10.1186/s12885-023-10655-2 (PMC10594864; doi:10.1186/s12885-023-10655-2)
Supplement: Supplementary file 3 — Additional file 3: Supplementary Fig. 3. Correlation of RMI2 and beta-catenin (CTNNB1) expression in the TCGA dataset. [file 12885_2023_10655_MOESM3_ESM.pdf]

Supplementary Figure 3.

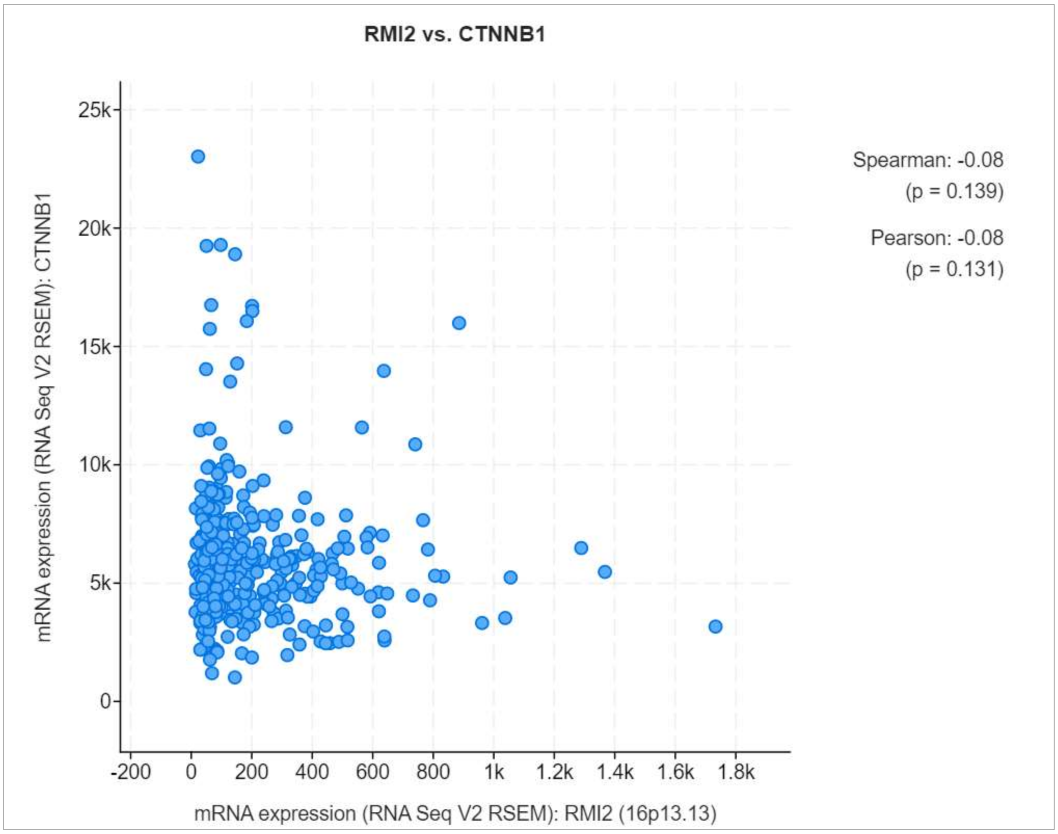

Correlation of RMI2 and beta-catenin (CTNNB1) expression in the TCGA dataset.
