# Supplementary material for: A combined bioinformatics and experimental approach identifies RMI2 as a Wnt/β-catenin signaling target gene related to hepatocellular carcinoma
Source: BMC Cancer. 2023 Oct 24;23:1025. doi: 10.1186/s12885-023-10655-2 (PMC10594864; doi:10.1186/s12885-023-10655-2)
Supplement: Supplementary file 4 — Additional file 4: Supplementary Fig. 4. The original images for western blot in article. [file 12885_2023_10655_MOESM4_ESM.pdf]

Fig 2A

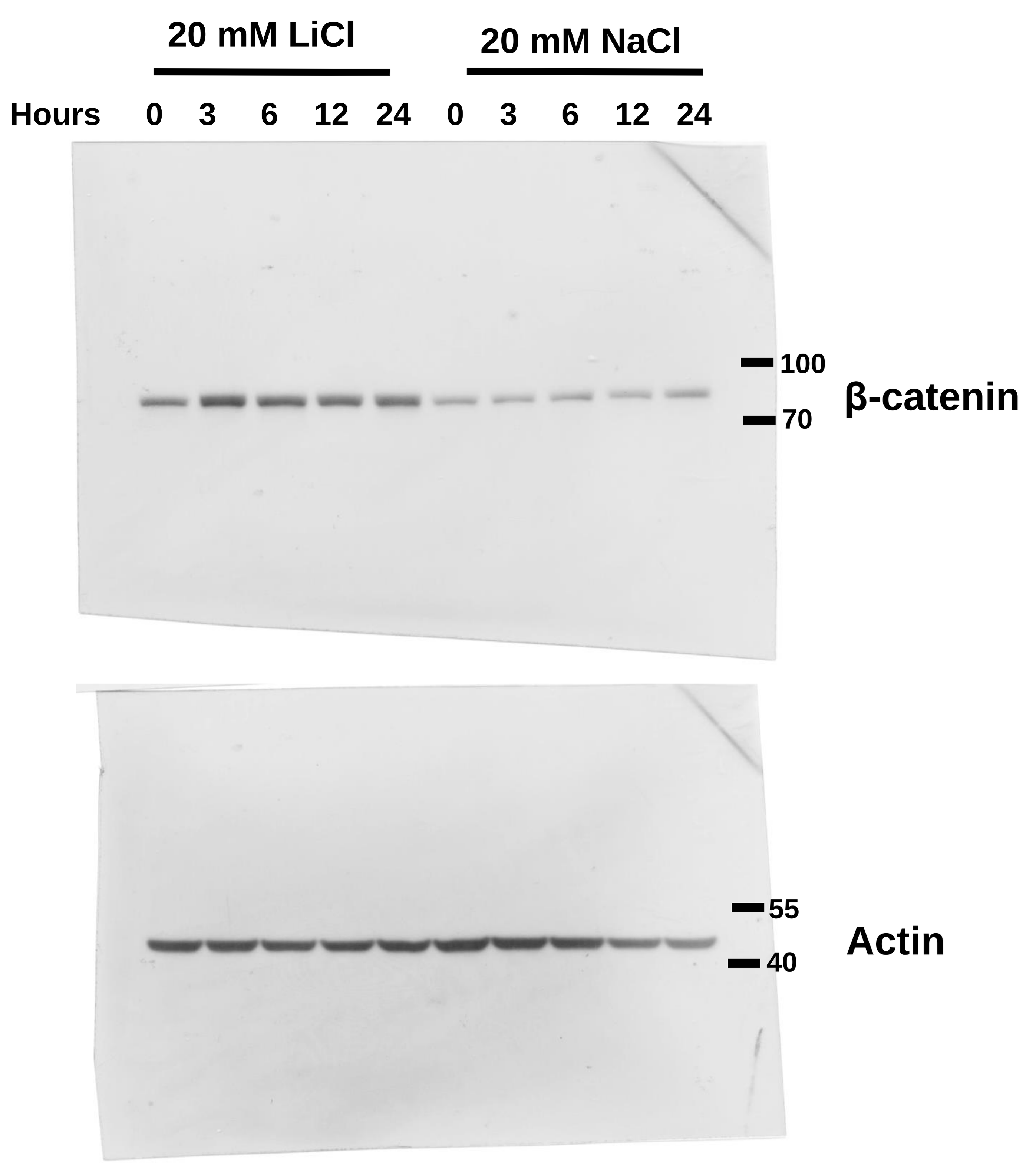

Fig 2B

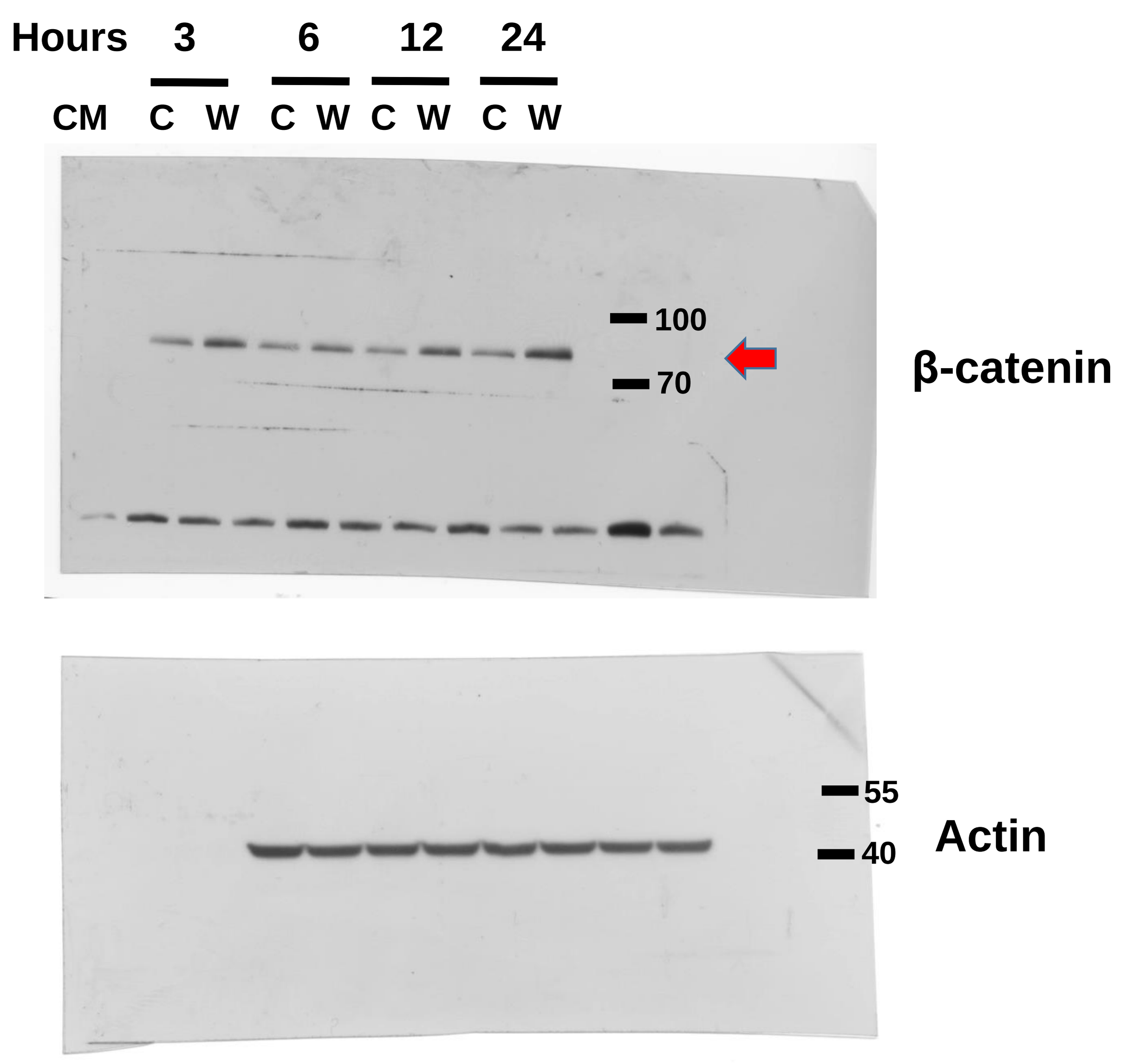

Fig 5A

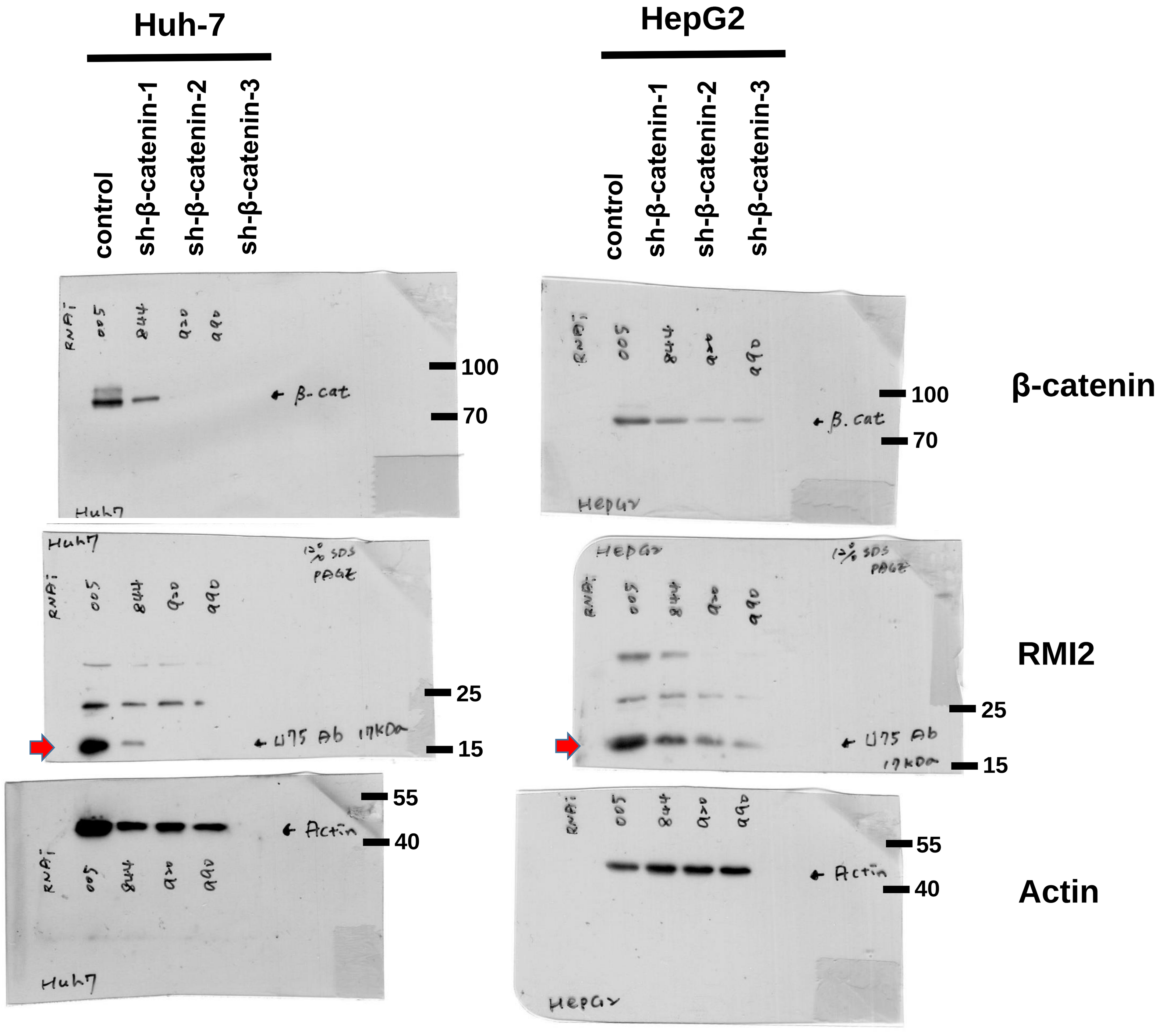

Fig 5B

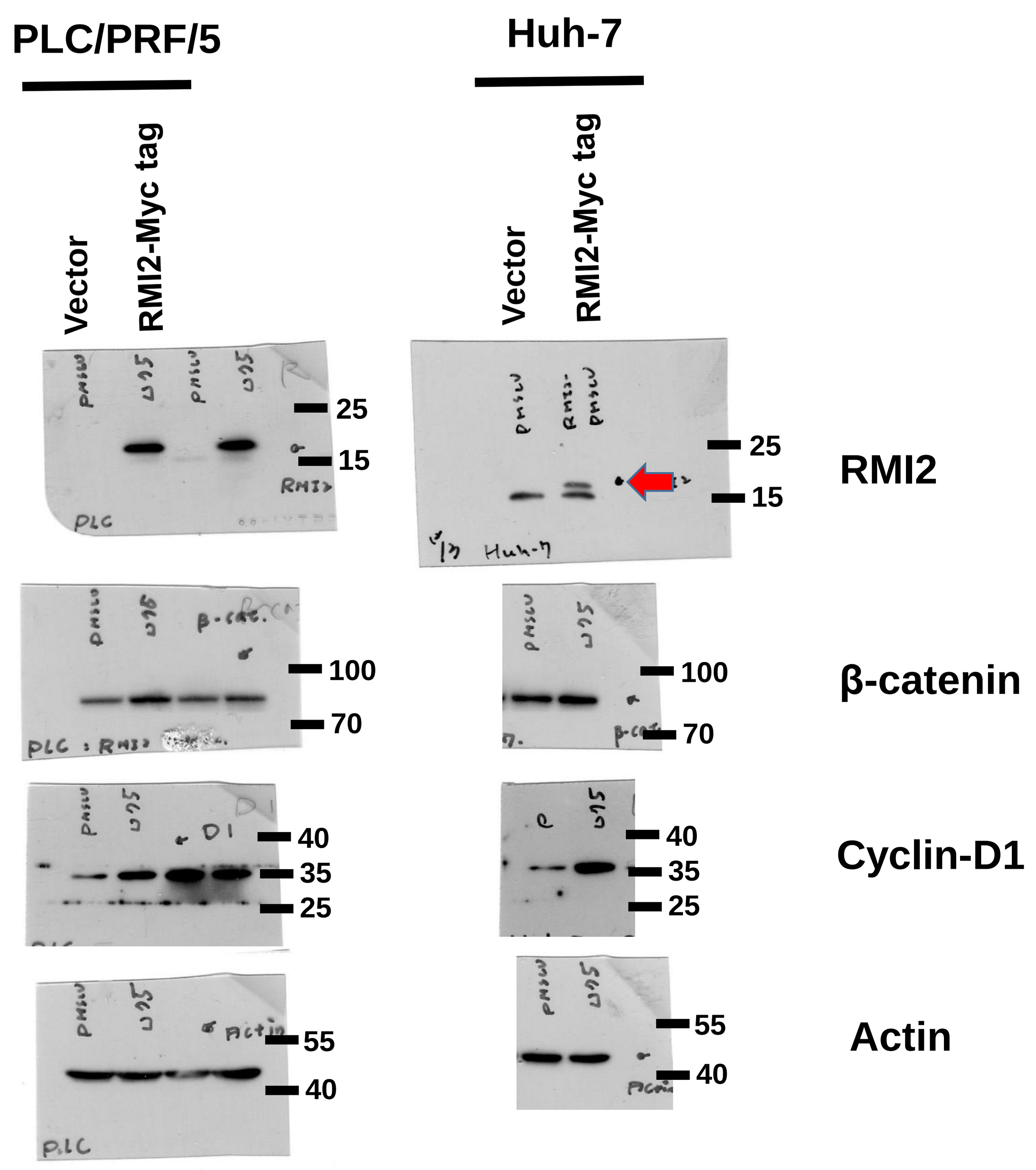

Supplementary Figure 4. The original images for western blot in article.

Fig 5C

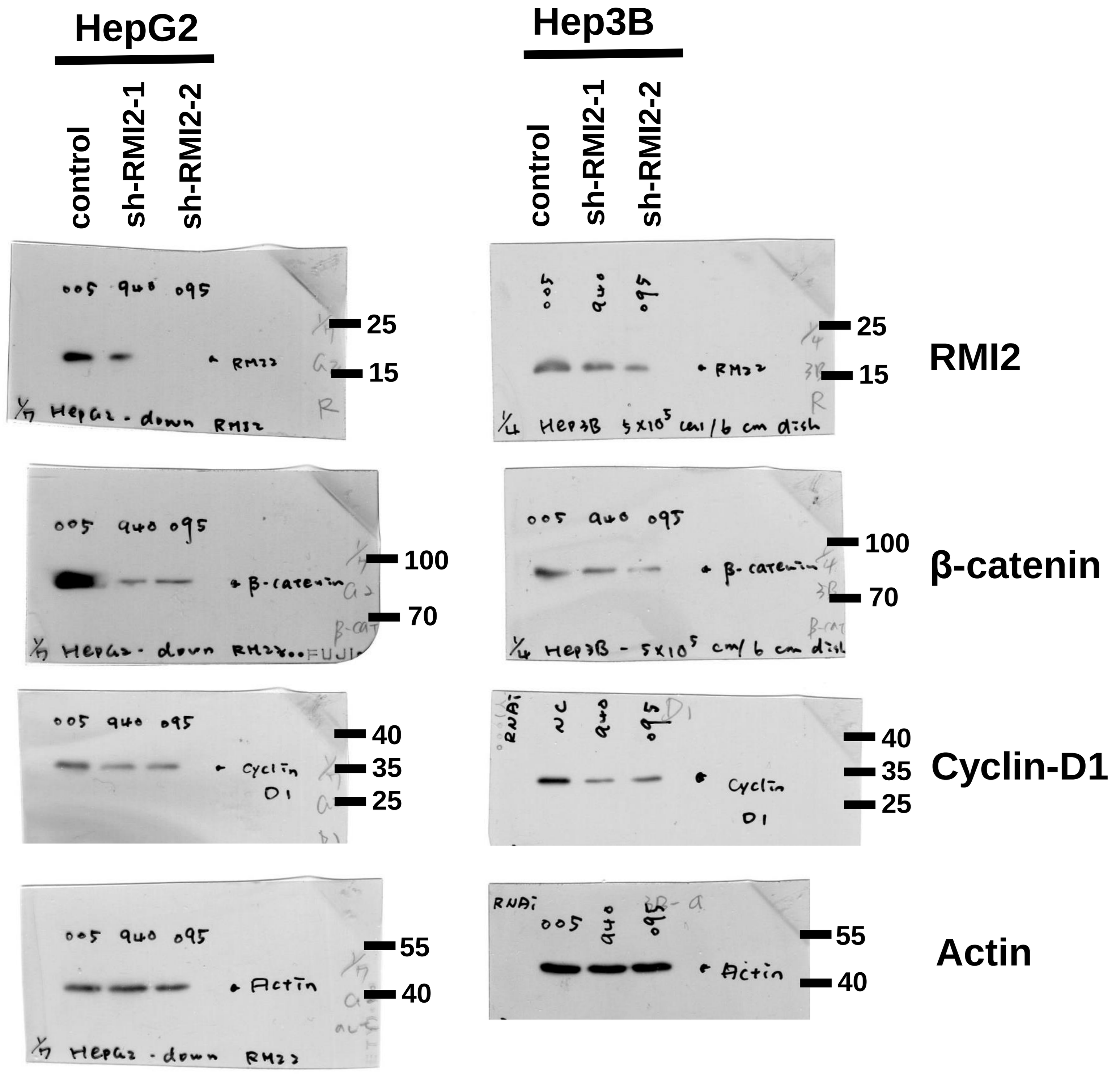

Fig 5F

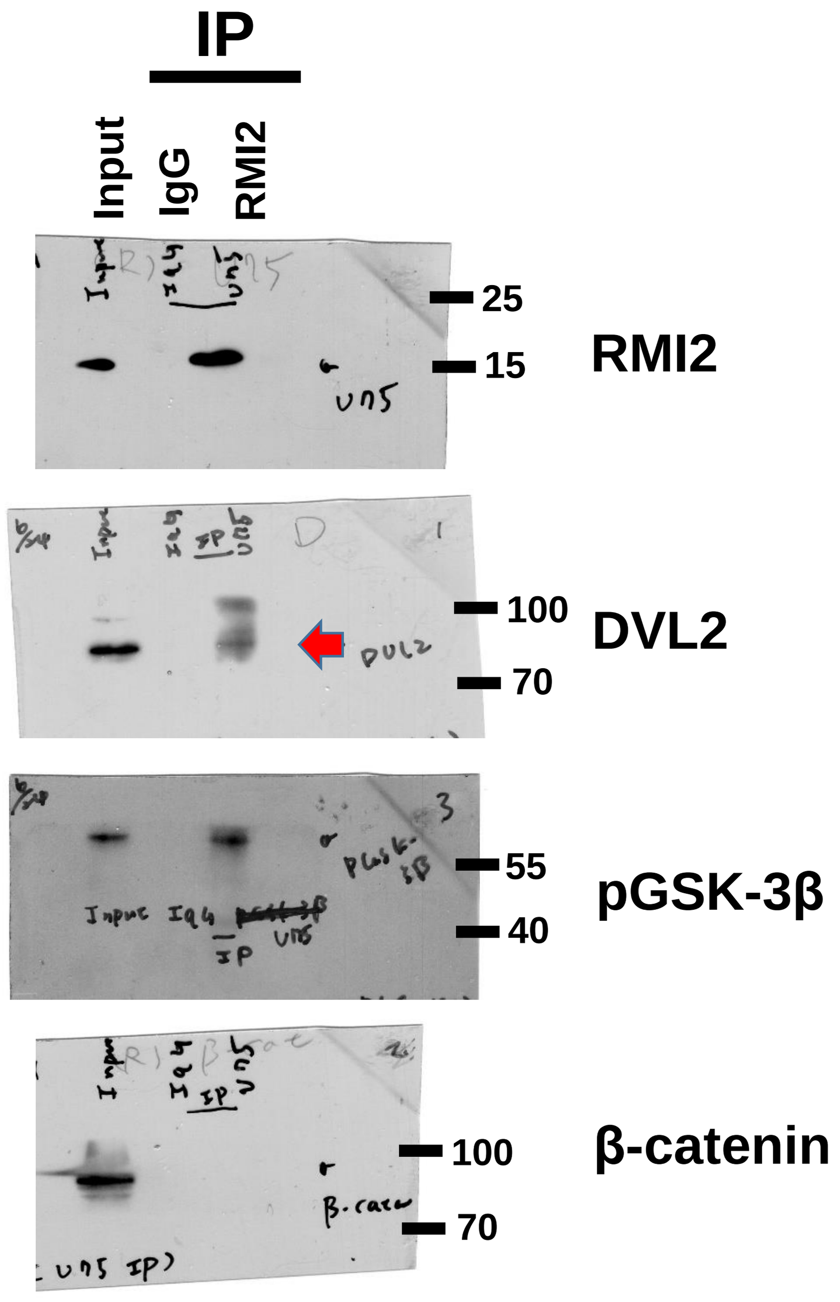

Fig 5G

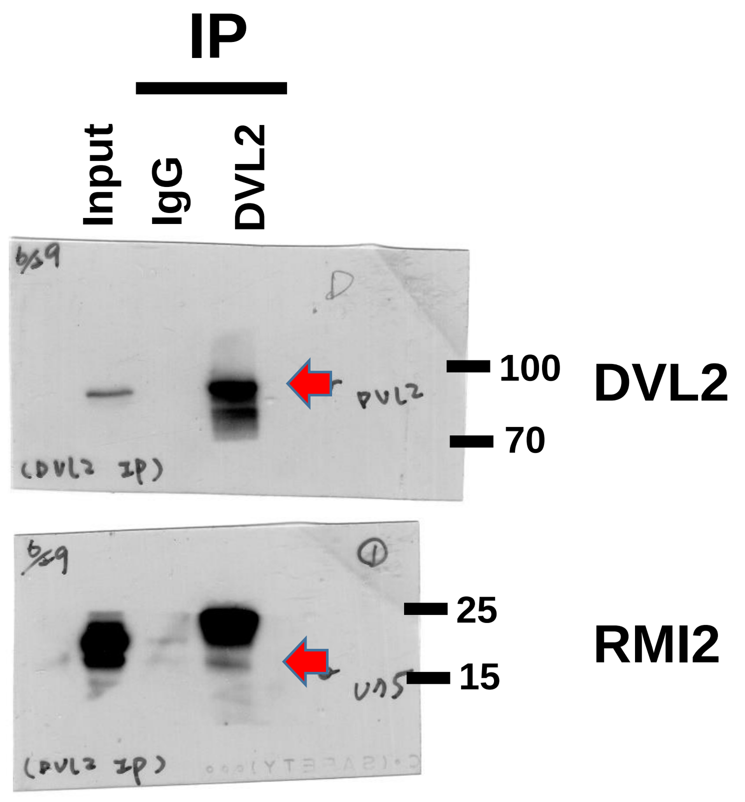

Fig 5H

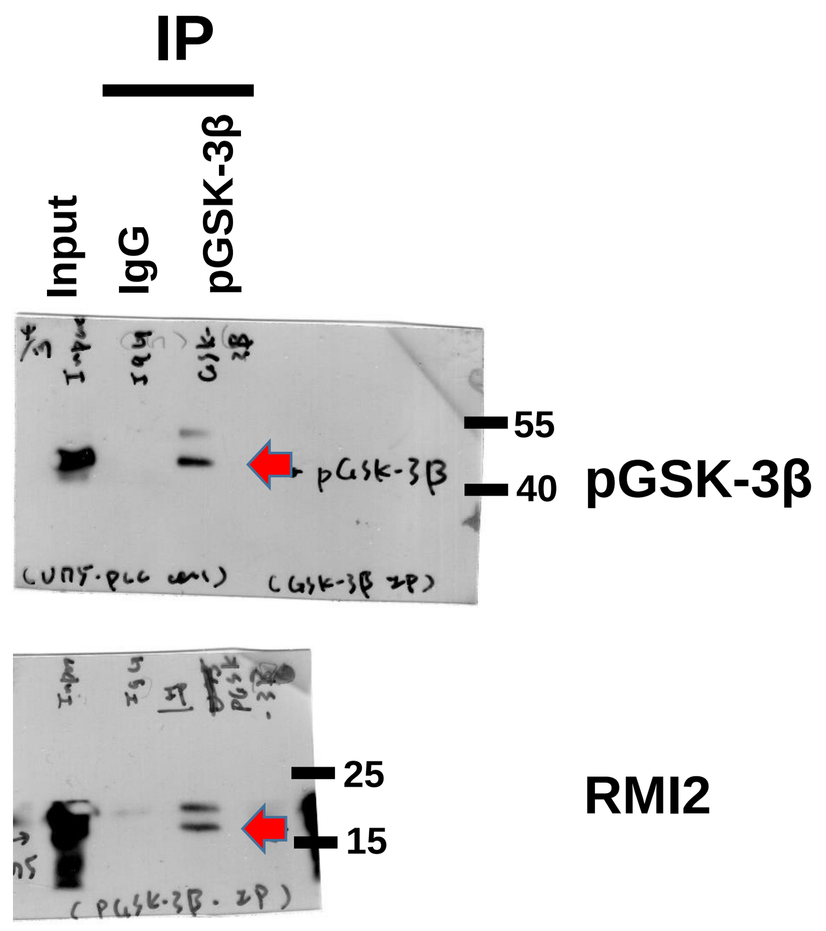

Supplementary Figure 4. The original images for western blot in article.

Fig. 7A -left

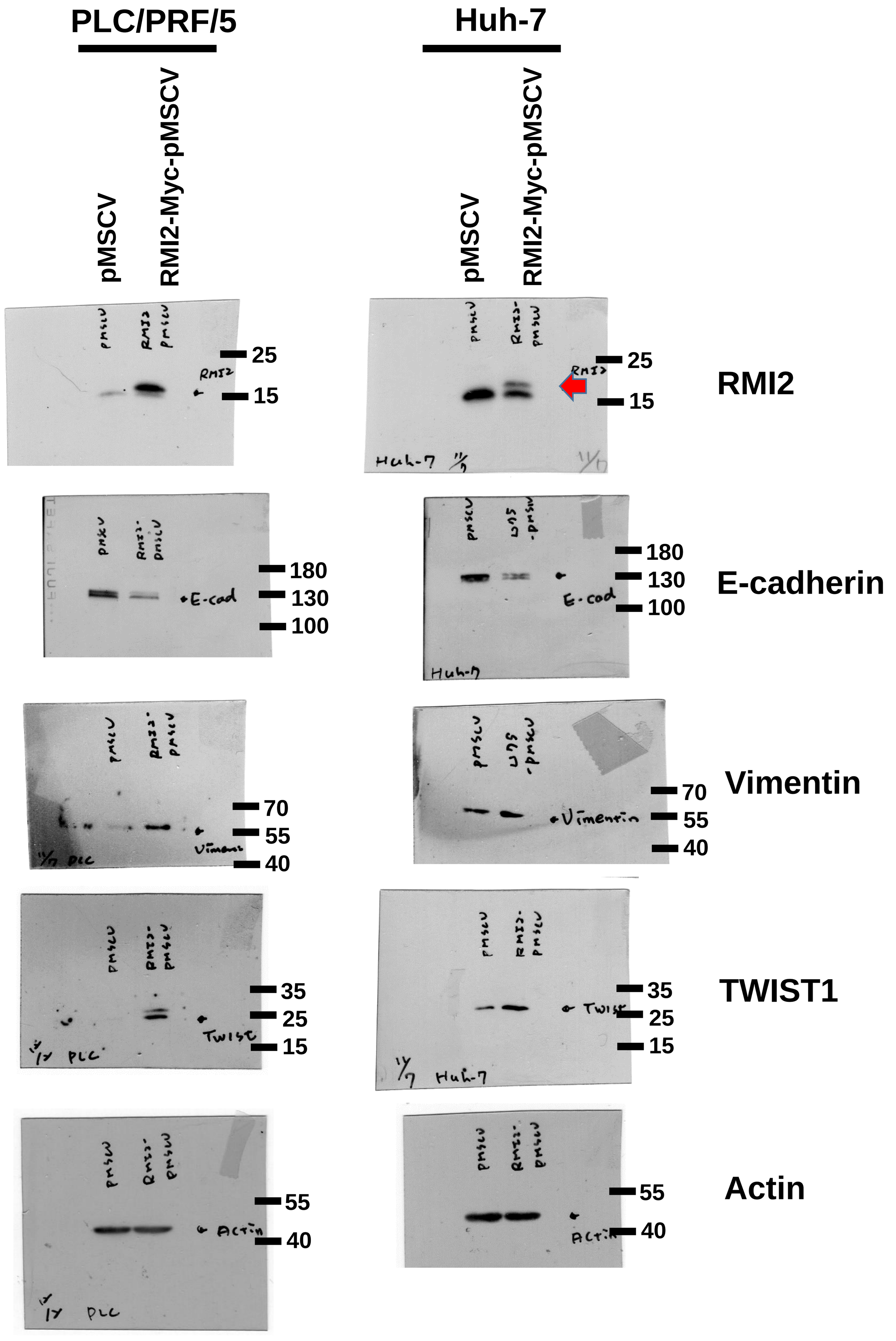

Fig. 7A - right

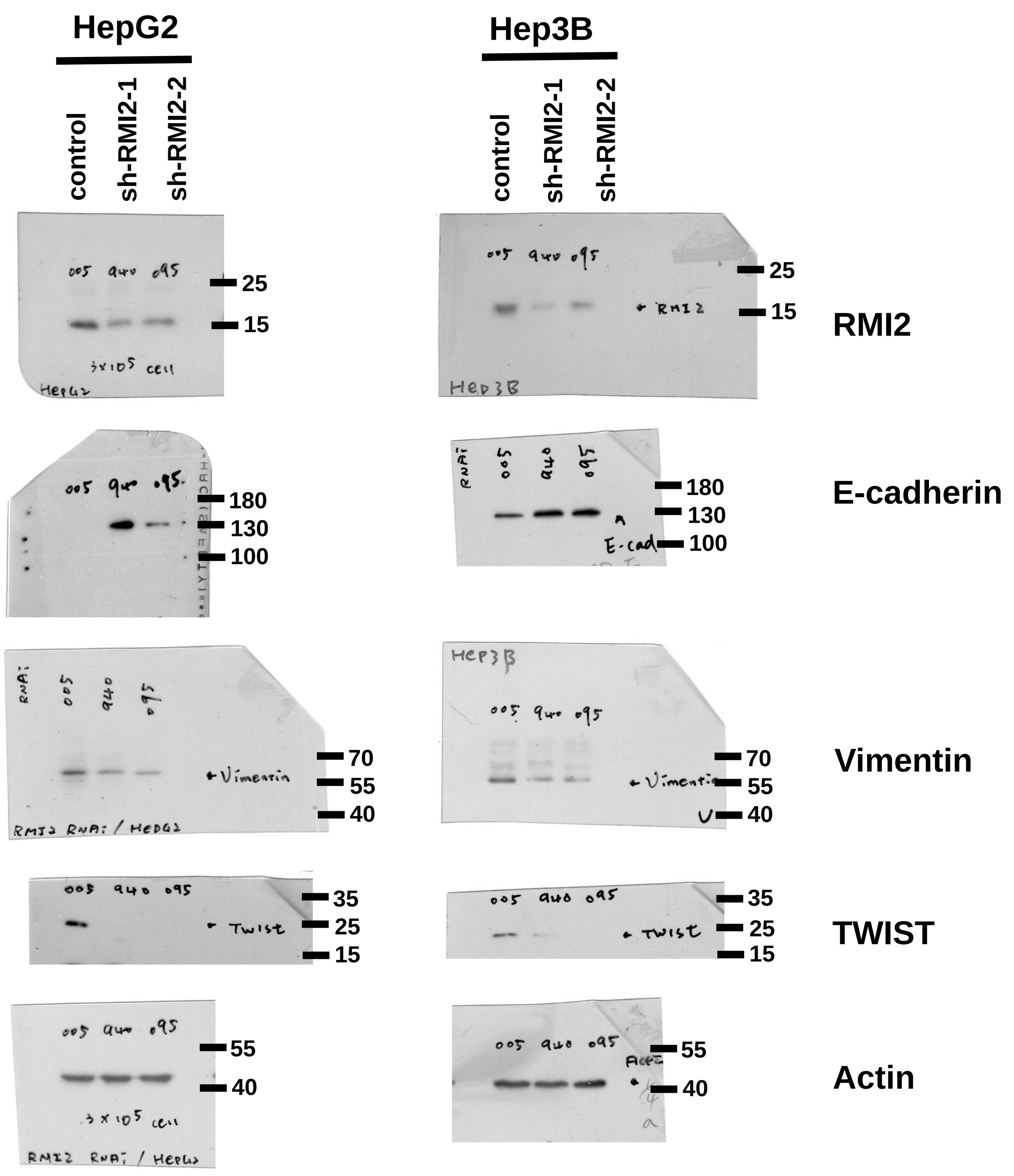

Fig 8D

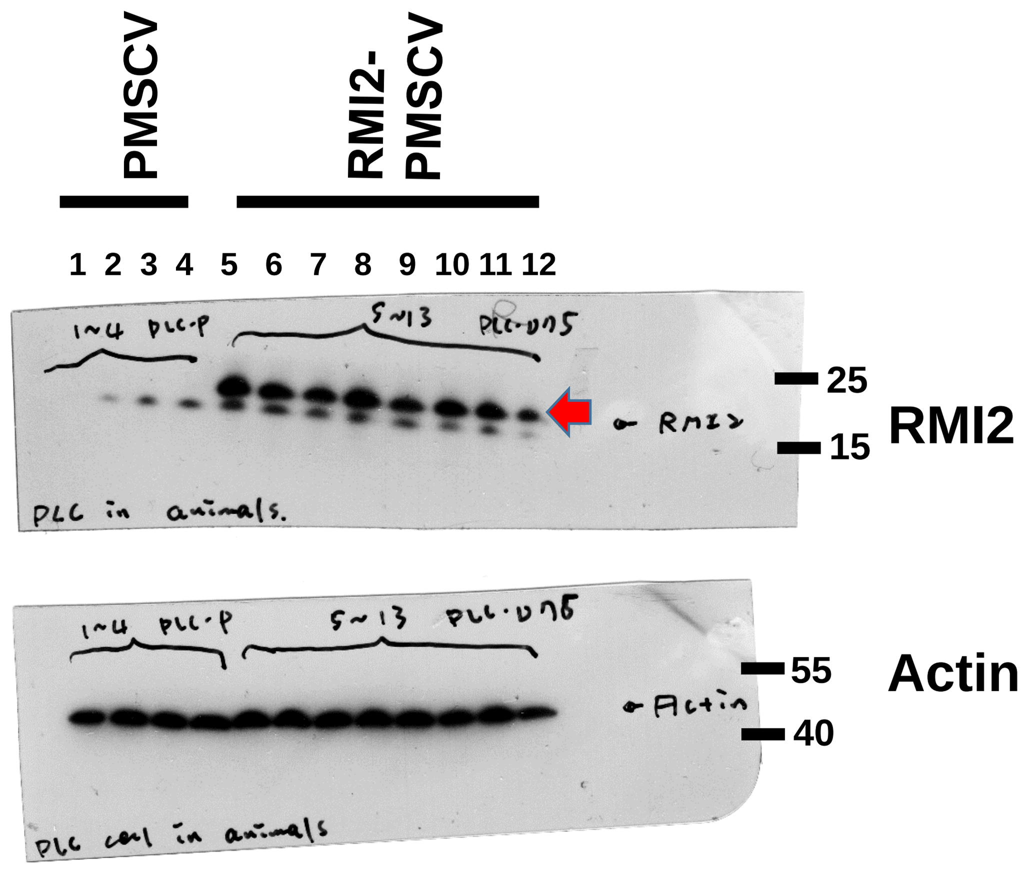

Supplementary Figure 4. The original images for western blot in article.
