# Supplementary material for: A combined bioinformatics and experimental approach identifies RMI2 as a Wnt/β-catenin signaling target gene related to hepatocellular carcinoma
Source: BMC Cancer. 2023 Oct 24;23:1025. doi: 10.1186/s12885-023-10655-2 (PMC10594864; doi:10.1186/s12885-023-10655-2)
Supplement: Supplementary file 5 — Additional file 5: Supplementary Fig. 5. The original images for agarose gel in article. [file 12885_2023_10655_MOESM5_ESM.pdf]

Figure 2A

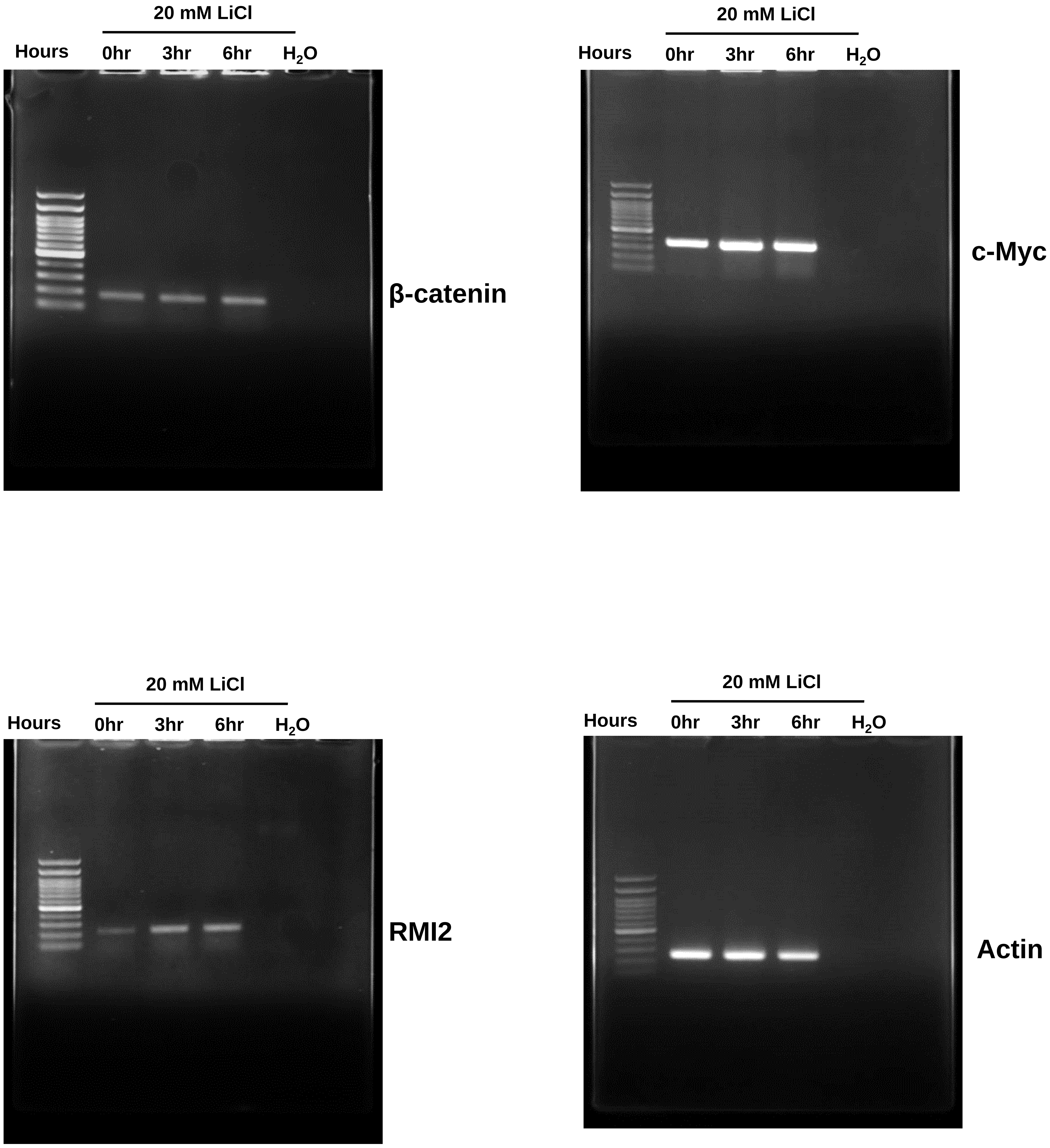

Supplementary Figure 5. The original images for agarose gel in article.

Figure 2B

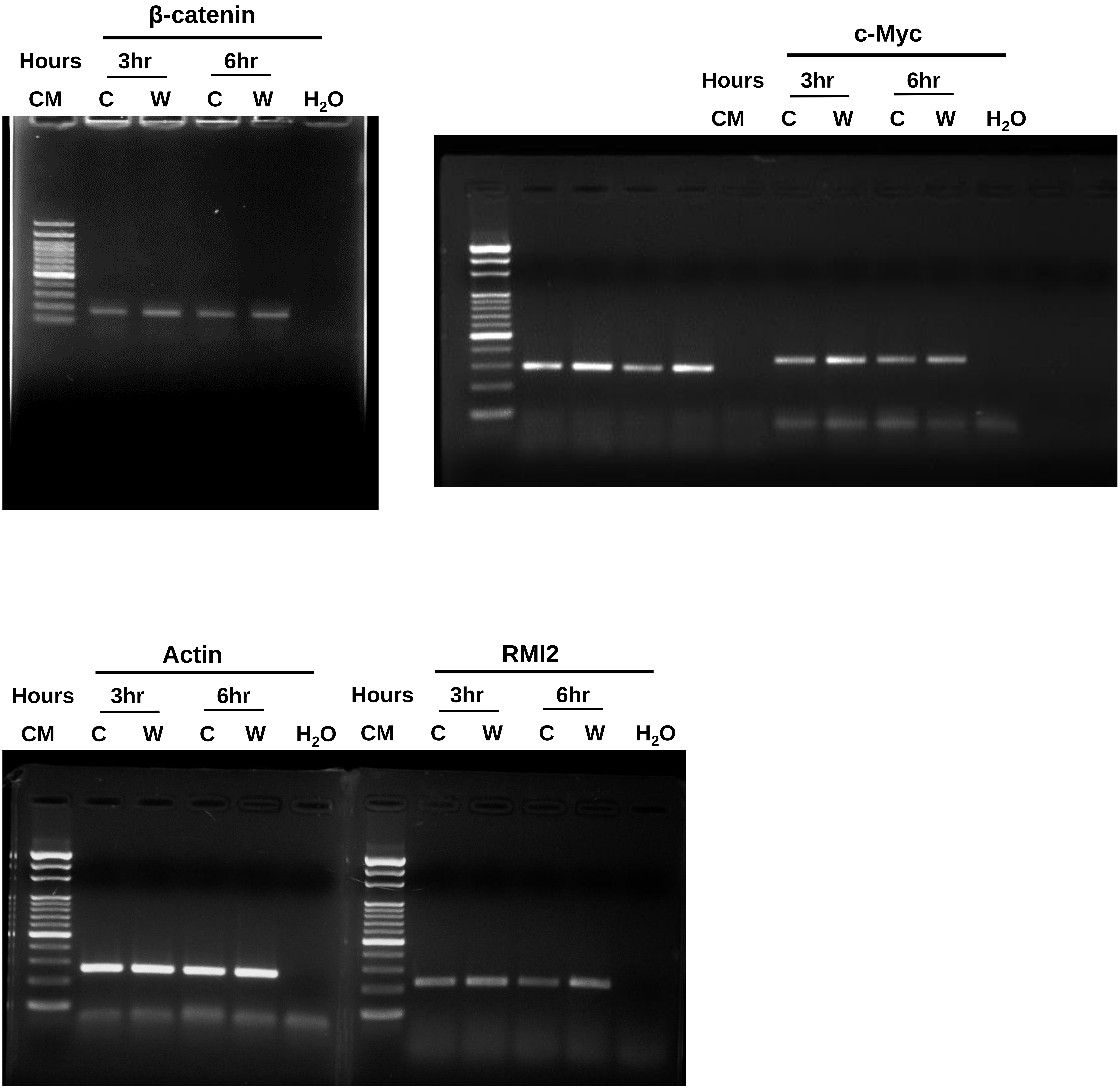

Supplementary Figure 5. The original images for agarose gel in article.

Figure 3AE

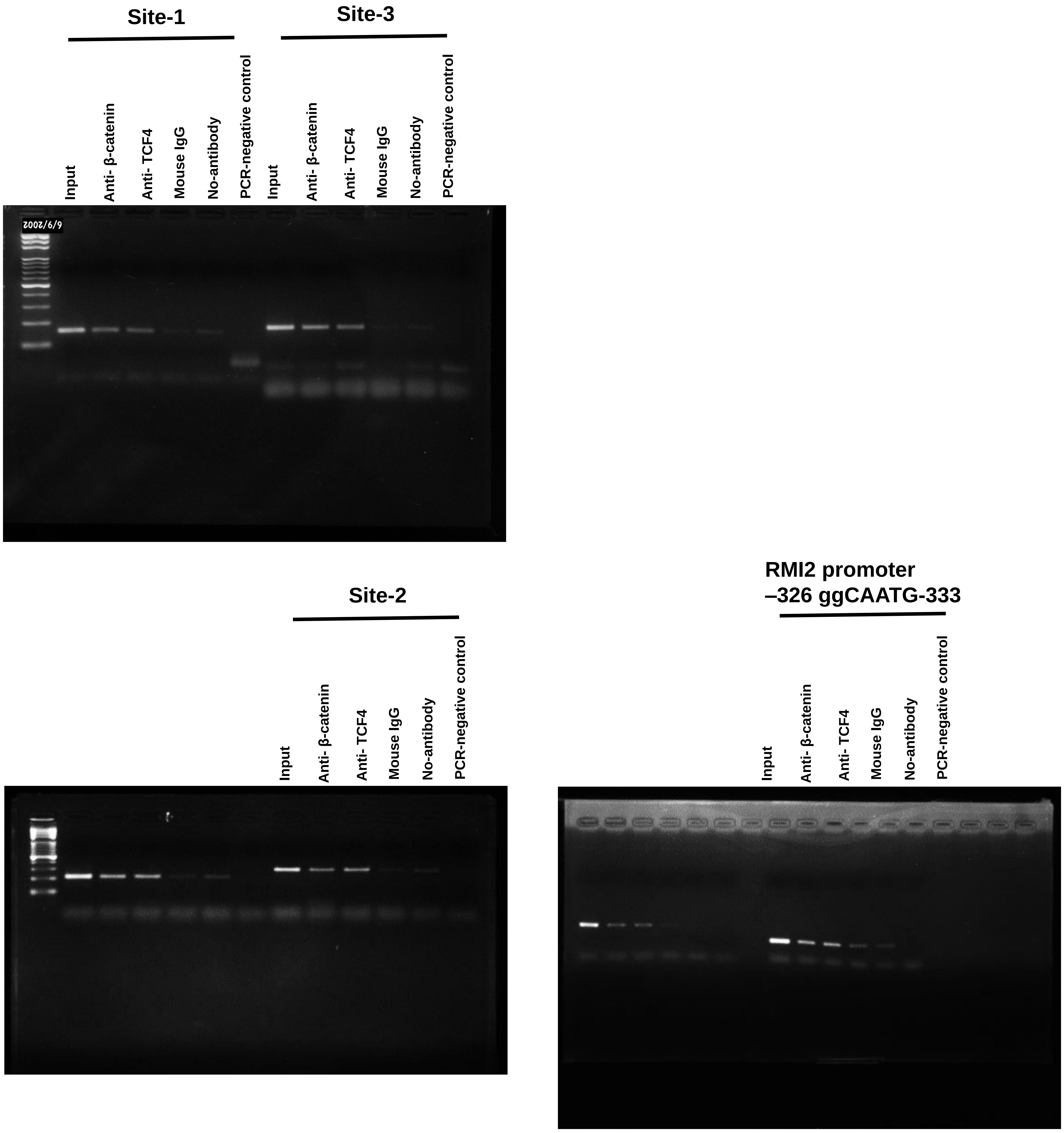

Supplementary Figure 5. The original images for agarose gel in article.
