# Supplementary material for: A combined bioinformatics and experimental approach identifies RMI2 as a Wnt/β-catenin signaling target gene related to hepatocellular carcinoma
Source: BMC Cancer. 2023 Oct 24;23:1025. doi: 10.1186/s12885-023-10655-2 (PMC10594864; doi:10.1186/s12885-023-10655-2)

# Steps to download X-crunch dataset.

1. Please click hyperlink [https://osf.io/pf6hr/files/osfstorage](https://osf.io/pf6hr/files/osfstorage).

2. If you want to download X-crunch.txt file, please click icon of red box A. If you want to download X-crunch.xlsx file, please click icon of red box B.

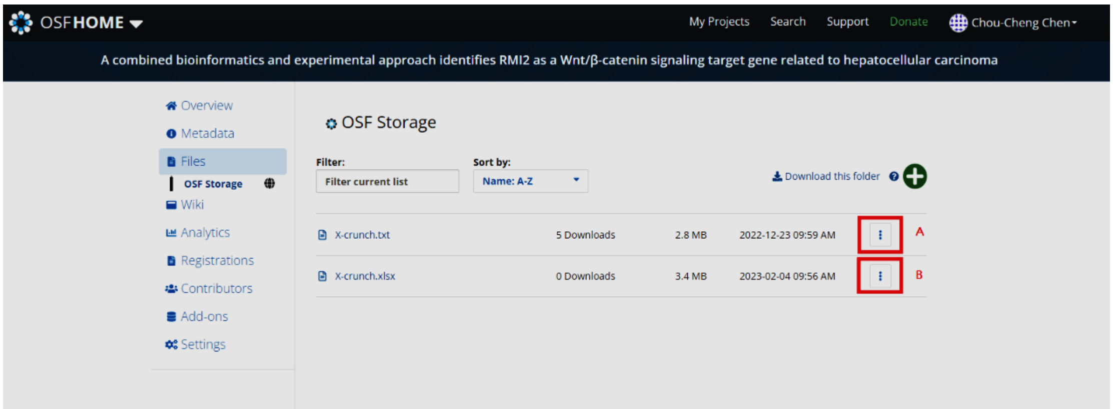


3. You will see download icon after step 2, and please click download icon to download file.

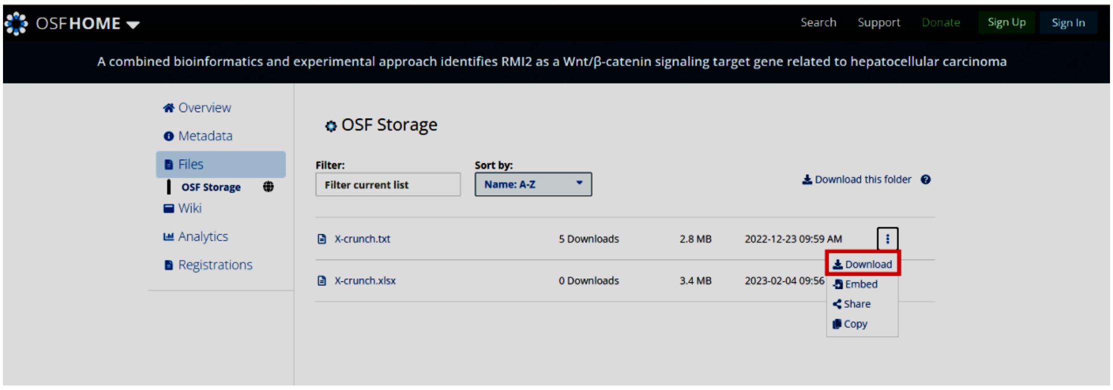

Supplement: Supplementary file 6 — Additional file 6. Steps to download X-crunch dataset. [file 12885_2023_10655_MOESM6_ESM.pdf]
