# Supplementary material for: A combined bioinformatics and experimental approach identifies RMI2 as a Wnt/β-catenin signaling target gene related to hepatocellular carcinoma
Source: BMC Cancer. 2023 Oct 24;23:1025. doi: 10.1186/s12885-023-10655-2 (PMC10594864; doi:10.1186/s12885-023-10655-2)
Supplement: Supplementary file 7 — Additional file 7: Supplementary Table 1. Target genes of Wnt/beta-catenin signaling from http://www.stanford.edu/group/nusselab/cgi-bin/wnt/. [file 12885_2023_10655_MOESM7_ESM.docx]

**Supplementary Table 1.** Target genes of Wnt/beta-catenin signaling from http://www.stanford.edu/group/nusselab/cgi-bin/wnt/

| Cyclin D | LBH | TNF family 41BB ligand | CTLA-4 | NOS2 | Proliferin |
| --- | --- | --- | --- | --- | --- |
| Tcf-1 | LGR5 | Twist 1 | BTEB2 | FGF20 | Emp |
| LEF1 | Sox9 | Twist 2 | versican | Gremlin | MDR1 |
| Axin2 | Sox17 | WISP-1 | Engrailed | RANK ligand | Cdx4 |
| EphB2 | Runx2 | WISP-2 | CD44 | Pituitary tumor transforming gene | EGFR |
| EphB3 | SALL4 | Proglucagon | autotaxin | FoxN1 | Eda |
| BMP4 | Cyr61 | Osteocalcin | ISLR | MMP 26 | Keratin |
| claudin-1 | Sox2 | Irx3 | Six3 | Nanog | FGF4 |
| survivin | Delta-like 1 | neurogenin 1 | c-myc | Fibronectin | ret |
| VEGF | Oct4 | NeuroD1 | n-myc | Wnt3a | Tnfrsf19 |
| FGF18 | Snail | Nkx2.2 | PPARdelta | Stra6 | Ubx |
| c-met | Frizzled 7 | IL-6 | c-jun | Wrch-1 | Dpp |
| endothelin-1 | Follistatin | periostin | fra-1 | Stromelysin | ITF-2 |
| Interleukin8 | Islet1 | betaTrCP | uPAR | Brachyury | CCN1 |
| Id2 | MMP 2 | sFRP-2 | MMP 7 | Cdx1 |  |
| Jagged1 | MMP 9 | Pitx2 | Nr-CAM | COX2 |  |
| Tiam1 | Connexin 43 | Retinoic acid receptor gamma | Gastrin | SP5 |  |
| Dickkopf | Connexin 30 | c-myc binding protein | Hath1 | Gbx2 |  |
| FGF9 | E-cadherin | P16ink4A | L1 neural adhesion | IGF |  |
