# Supplementary material for: A combined bioinformatics and experimental approach identifies RMI2 as a Wnt/β-catenin signaling target gene related to hepatocellular carcinoma
Source: BMC Cancer. 2023 Oct 24;23:1025. doi: 10.1186/s12885-023-10655-2 (PMC10594864; doi:10.1186/s12885-023-10655-2)
Supplement: Supplementary file 8 — Additional file 8: Supplementary Table 2. (A) Primer sequence used for PCR assays. (B) Primer sequence used for ChIP assays. (C) Primer sequence used for site-directed mutagenesis assays. [file 12885_2023_10655_MOESM8_ESM.docx]

**Supplementary Table 2.**

**(A) Primer sequence used for PCR assays**

| **Gene name** | **Primer name** | **Sequence** | **Size** |
| --- | --- | --- | --- |
| **For RT-PCR**  β-catenin | β-catenin -F | AGCCAATGGCTTGGAATGA | 174 bp |
|  | β-catenin -R | GGTGGTGGCCACCCATCT |  |
| c-Myc | c-Myc -F | GCAAGACTCCAGCGCCTTCTCTC | 364 bp |
|  | c-Myc -R | TGACACTGTCCAACTTGACCCTCTT |  |
| RMI2 | RMI2-F | GCTGTGAAGATGACAGACC | 217 bp |
|  | RMI2-R | CAAAGCAAGGCTCTCTGA |  |
| β-actin | β-actin-F | CATGTACGTTGCTATCCAGGC | 298 bp |
|  | β-actin-R | CTCCTTAATGTCACGCACGAT |  |

**(B) Primer sequence used for ChIP assays**

| **Gene name** | **Primer name** | **Sequence** | **Size** |
| --- | --- | --- | --- |
| **For ChIP assay** |  |  |  |
| RMI2-TBE1 | RMI2-pro3-F | AGCAGATGGATATTTGCTGA | 176 bp |
|  | RMI2-pro3-R | CAAGGTGGTCTGGAACTC |  |
| RMI2-TBE2/3/4 | RMI2-pro2-F | GCTGAATACTCTCTGAATGC | 286 bp |
|  | RMI2-pro2-R | CGCCCAGCTAATTTTTTGT |  |
| RMI2-TBE5/6 | RMI2-pro1-F | GCTTCCTTAAGCCTCAGTTT | 202 bp |
|  | RMI2-pro1-R | CTCCTGACCTCGTGATCT |  |
|  |  |  |  |

**(C) Primer sequence used for site-directed mutagenesis assays**

| **Gene name** | **Primer name** | **Sequence** |
| --- | --- | --- |
| **For site-directed mutagenesis** | | |
| RMI2-TBE1 | RMI2-TBE1-PM-F | GTAATCCCAGCGAATTCGGAGGCCAAGGAGGC |
|  | RMI2-TBE1-PM-R | GCCTCCTTGGCCTCCGAATTCGCTGGGATTAC |
| RMI2-TBE1 | RMI2-TBE1-PM-mu-F | TAATTACCTTGACTTTAGCTTGACATTTGGTTGTTGG |
|  | RMI2-TBE1-PM-mu-R | CCAACAACCAAATGTCAAGCTAAAGTCAAGGTAATTA |
| RMI2-TBE2 | RMI2-TBE2-mu-F | CCTCGTCTCTACTAAAAGCTAGTACAAAAAATTAGCTGGGCG |
|  | RMI2-TBE2-mu-R | CGCCCAGCTAATTTTTTGTACTAGCTTTTAGTAGAGACGAGG |
|  |  |  |
